# Trafficking of cholesterol to the ER is required for NLRP3 inflammasome activation

**DOI:** 10.1083/jcb.201709057

**Published:** 2018-10-01

**Authors:** Marianne de la Roche, Claire Hamilton, Rebecca Mortensen, A. Arockia Jeyaprakash, Sanjay Ghosh, Paras K. Anand

**Affiliations:** 1 Infectious Diseases and Immunity, Department of Medicine, Imperial College London, London, UK; 2 Wellcome Trust Centre for Cell Biology, Institute of Cell Biology, University of Edinburgh, Edinburgh, UK; 3 Department of Biochemistry, University of Cambridge, Cambridge, UK

## Abstract

Cellular lipids determine membrane integrity and fluidity and are being increasingly recognized to influence immune responses. Cellular cholesterol requirements are fulfilled through biosynthesis and uptake programs. In an intricate pathway involving the lysosomal cholesterol transporter NPC1, the sterol gets unequally distributed across intracellular compartments. By using pharmacological and genetic approaches targeting NPC1, we reveal that blockade of cholesterol trafficking through the late endosome–lysosome pathway blunts NLRP3 inflammasome activation. Altered cholesterol localization at the plasma membrane (PM) in *Npc1^−/−^* cells abrogated AKT–mTOR signaling by TLR4. However, the inability to activate the NLRP3 inflammasome was traced to perturbed cholesterol trafficking to the ER but not the PM. Accordingly, acute cholesterol depletion in the ER membranes by statins abrogated casp-1 activation and IL-1β secretion and ablated NLRP3 inflammasome assembly. By contrast, assembly and activation of the AIM2 inflammasome progressed unrestricted. Together, this study reveals ER sterol levels as a metabolic rheostat for the activation of the NLRP3 inflammasome.

## Abstract (precis)

Cellular lipid metabolism is being increasingly recognized to influence inflammatory responses. de la Roche et al. reveal that cellular sterol trafficking to the endoplasmic reticulum is required for the assembly and the activation of the NLRP3 inflammasome, thereby coupling lipid homeostasis to innate immune signaling.

## Introduction

The inflammasome is a multiprotein complex that plays critical roles in infectious, inflammatory, and autoimmune diseases. The NLRP3 inflammasome is the most characterized inflammasome in terms of the diverse stimuli that are known to activate it. Activation of the NLRP3 inflammasome requires assembly of NLRP3 and caspase-1 (casp-1) bridged together through the adaptor protein ASC, wherein casp-1 undergoes autoproteolytic processing. Subsequently, active casp-1 cleaves precursor forms of cytokines interleukin (IL)–1β and IL-18, which can then be secreted (Man and Kanneganti, 2015; Hamilton et al., 2017). Casp-1 also cleaves gasdermin D (GSDMD), making its N-terminal pore-forming domain active, leading to cell rupture (Kayagaki et al., 2015; Shi et al., 2015). Distinct exogenous, endogenous, and environmental stimuli are known to activate the NLRP3 inflammasome, implying that these stimuli do not bind NLRP3 directly but likely converge on shared upstream pathways. The mechanistic details of NLRP3 activation remain ambiguous.

Lipids are known to carry out diverse functions within cells, including being a major component of cell membranes, and as signaling messengers. Cholesterol is an essential lipid in mammalian cell membranes aiding varied functions, the most fundamental of which are membrane integrity and fluidity (Maxfield and Tabas, 2005). Levels of cholesterol in the cell are maintained through de novo synthesis in the ER, and uptake of low-density lipoproteins (LDLs) derived from dietary cholesterol. Excess free cholesterol can be toxic to cells; thus, sterol homeostasis needs to be integrated by a combination of cholesterol uptake, biosynthesis, and efflux programs. At the subcellular level, cholesterol follows an intricate pathway in cells (Ikonen, 2008). Exogenously obtained LDL bound to LDL receptor is internalized at the plasma membrane (PM) and is transported through the endocytic pathway to the late endosomes–lysosomes, where cholesterol esters within the LDL core are hydrolyzed by acid lipases. Unesterified or free cholesterol translocates through the lysosomal cholesterol transporter Niemann-Pick C1 (NPC1) to other cellular sites such as the PM and the ER. In the ER, cholesterol can be reesterified, permitting cytoplasmic storage in the form of lipid droplets.

Until recently, cholesterol has mostly been accepted to have an influence on immunity during pathological conditions such as in atherosclerosis (Fessler, 2016). However, evidence suggests that homeostatic lipid metabolism and trafficking directly regulate the inflammatory pathways in macrophages. For example, defective lipid trafficking in the absence of NPC1 leads to the lysosomal storage disorder Niemann-Pick disease (Platt et al., 2012). Mutations in the cholesterol efflux transporter, ABCA1, give rise to signs and symptoms of Tangier disease (Fasano et al., 2012). Similarly, perturbations in lipid metabolism contribute to several human pathologies including cardiovascular, obesity, and neurodegenerative diseases (Maxfield and Tabas, 2005). In addition to contributing to the pathogenesis of several diseases, cholesterol is also exploited by pathogens for their entry and proliferation within host cells. Several pathogens that lack the capacity for de novo sterol synthesis use cholesterol for their survival and replication by either increasing host lipid biosynthesis or redirecting cholesterol transport pathways (Coppens et al., 2000; Lauer et al., 2000; Carabeo et al., 2003; Kaul et al., 2004; Ilnytska et al., 2013). These studies suggest that reducing lipid synthesis may serve to limit nutrients available to pathogens, thus benefitting host cells. Conversely, host cells need lipids for mounting a robust immune response to infection through conserved pattern recognition receptors (Castrillo et al., 2003; York et al., 2015). Together, these studies lead to the hypothesis that lipid homeostasis is critical for an effective inflammatory response with implications for homeostatic lipid trafficking in both infectious and inflammatory diseases. Whether perturbations in homeostatic cholesterol trafficking pathway impact inflammasome activation remains unknown.

In this study, by using pharmacological and genetic tools, we demonstrate that selective perturbation of the cellular cholesterol trafficking in macrophages ablates inflammasome activation. Mechanistically, perturbed sterol trafficking in *Npc1* deficiency leads to two distinct effects: altered PM cholesterol levels resulted in inhibition of the AKT–mTOR pathway, while reduced cholesterol trafficking to the ER blunted NLRP3 inflammasome assembly. Accordingly, acute cholesterol depletion in the ER by statins decreased IL-1β secretion, which could be restored by supplementing with exogenous cholesterol. Our findings thus implicate sterol synthesis and distribution as critical factors influencing the activation of the inflammasome, thereby coupling lipid homeostasis to innate immune signaling.

## Results

### Lysosomal sterol accumulation dampens inflammasome activation

**Figure 1.**
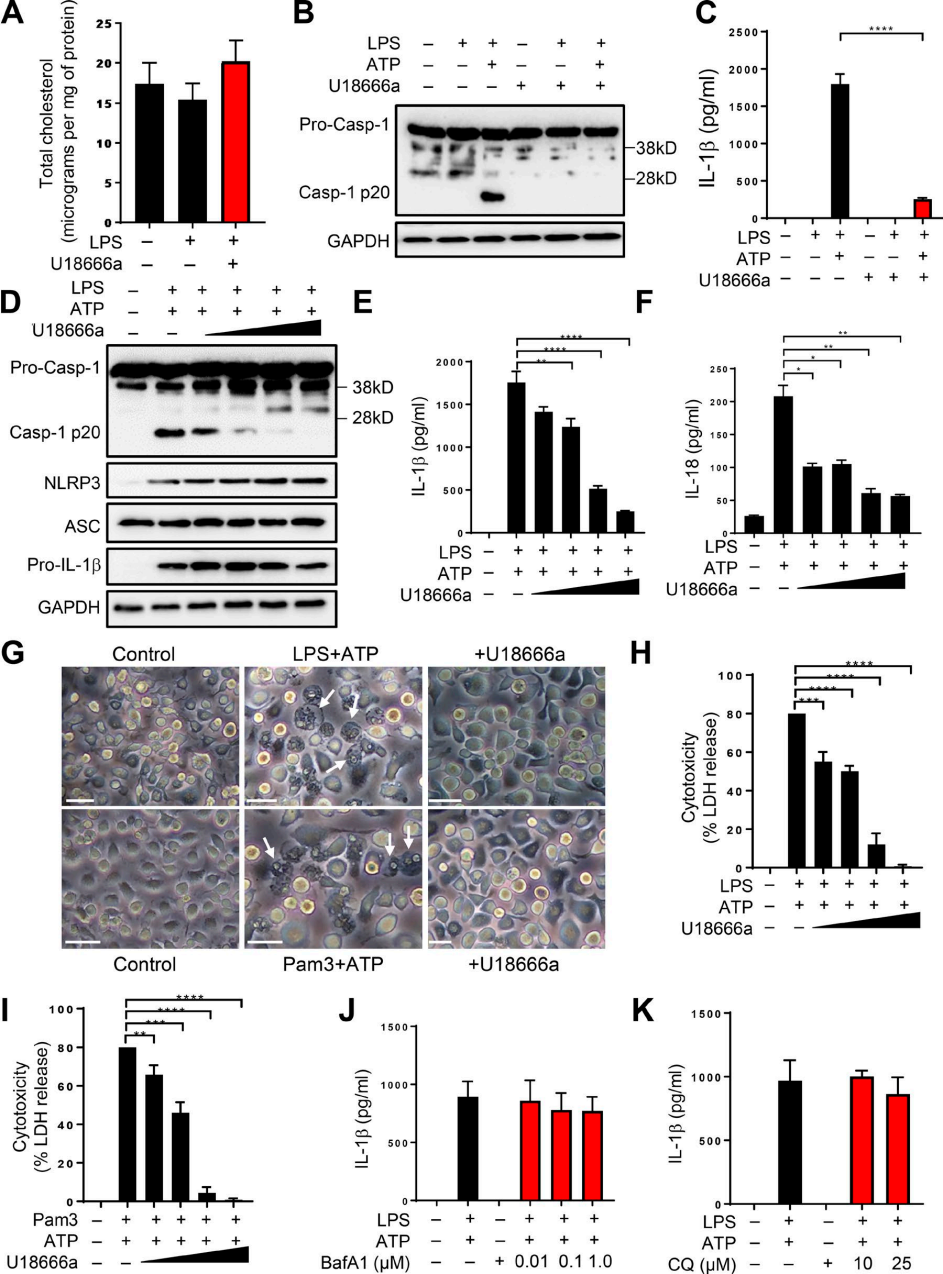
**Lysosomal sterol accumulation dampens inflammasome activation. (A)** BMDMs were incubated with U18666a in the presence or absence of LPS followed by measurement of total cholesterol. **(B)** BMDMs were either left untreated or exposed to 5 µg/ml U18666a before treating them with LPS (500 ng/ml; 4 h) and ATP (5 mM; 45 min). Cell lysates were immunoblotted for casp-1 antibody, and GAPDH was used as loading control. **(C)** IL-1β release from cells treated as above. **(D)** BMDMs were either left untreated or exposed to increasing concentrations of U18666a (1, 2, 5, and 10 µg/ml) before stimulating them with LPS and ATP. **(E and F)** Cell lysates were immunoblotted for the antibodies indicated, and cell supernatants were analyzed for IL-1β (E) or IL-18 (F) by ELISA. **(G)** Microscopy images of cells treated as above in B or with Pam3 (500 ng/ml; 4 h) followed by ATP. Arrows show characteristic pyroptotic cell death. Bars, 20 µm. **(H and I)** LDH release in supernatants from cells treated as in G. **(J and K)** LPS-primed BMDMs treated with indicated concentrations of Baf A1 (J) or CQ (K) followed by ATP. Data shown are mean ± SD, and experiments shown are representative of at least three independent experiments. *, P < 0.05; **, P < 0.01; ***, P < 0.001; ****, P < 0.0001, by Student’s *t* test.

Homeostatic cholesterol trafficking is important as its distribution among subcellular organelles upholds vital housekeeping functions, and is critically dependent on lysosomal cholesterol transporter NPC1. The lysosomal egress of cholesterol can be blocked by exposing cells to a cationic amphiphile and an androstenolone derivative, U18666a, which specifically targets NPC1 (Lu et al., 2015). To assess the effect of U18666a on cholesterol transport in our experiments, we first exposed bone marrow–derived mouse macrophages (BMDMs) and immortalized BMDMs (iBMDMs) to the compound and examined cholesterol localization by staining with a naturally fluorescent polyene antibiotic, filipin. Filipin specifically binds to free cholesterol in biological membranes and can be excited by UV fluorescence. Filipin staining of control cells revealed diffused cholesterol staining throughout the cell (Fig. S1). By contrast and in conformity with previous studies in different cell types, exposure of BMDMs and iBMDMs to U18666a resulted in punctate structures reminiscent of lysosomes, thus demonstrating lysosomal cholesterol accumulation (Fig. S1, A and B; Strauss et al., 2010; Maxfield and Wüstner, 2012). Notably, these punctate structures were completely absent in control cells. Furthermore, we observed no significant difference in total cholesterol levels in untreated and lipopolysaccharide (LPS)–treated cells in the presence or absence of U18666a, suggesting that the elevated cholesterol accumulation in lysosomes is a result of altered sterol trafficking (Fig. 1 A). Together, these results established lysosomal cholesterol accumulation in mouse macrophages exposed to U18666a.

Next, to investigate the impact of cellular sterol trafficking on inflammasome activation, we exposed control and U18666a-treated BMDMs to LPS and ATP stimulation, a canonical ligand for activating the NLRP3 inflammasome. Activation of the NLRP3 inflammasome results in cleavage of pro–casp-1 to its active form, which subsequently processes precursor forms of cytokines IL-1β and IL-18 to their biologically active forms. Exposure of LPS-primed control cells to ATP resulted in robust casp-1 activation, which was significantly abolished in cells where cholesterol distribution was restricted to lysosomes (Fig. 1 B). In agreement, this was accompanied with significant reduction in IL-1β secretion compared with control cells (Fig. 1 C). To assess the specificity of this response, we next performed these experiments in BMDMs exposed to a range of U18666a concentrations followed by LPS and ATP stimulation. In confirmation with our above results, this resulted in a dose-dependent decrease in both casp-1 activation and secretion of inflammasome-dependent cytokines IL-1β and IL-18 (Fig. 1, D–F). Finally, we confirmed these results with itraconazole, an antifungal drug that retards cholesterol trafficking from lysosomes. Similar to the results with U18666a, exposure to itraconazole resulted in reduced casp-1 activation and IL-1β secretion (Fig. S1 C). The precise mechanism by which itraconazole blocks cholesterol trafficking is not known. However, previous research established filipin colocalization with lysosomes but not PM, ER, and mitochondrial markers in endothelial cells exposed to the antifungal agent (Xu et al., 2010). Overall, these studies validated defective cholesterol trafficking as the cause of dampened inflammasome activation.

Activation of inflammasome is characterized by cell swelling and subsequent osmotic lysis in the form of pyroptotic cell death following GSDMD cleavage (Kayagaki et al., 2015; Shi et al., 2015). In control cells primed with either LPS or Pam3, stimulation with ATP caused pyroptotic cell death. However, upon exposure to U18666a, cell death, and thus the secreted levels of cytosol-localized cell death marker, lactate dehydrogenase (LDH), were markedly diminished (Fig. 1, G–I). Overall these results demonstrate reduced casp-1 activation, IL-1β and IL-18 secretion, and decreased pyroptosis when cellular cholesterol trafficking is inhibited.

### Impaired cholesterol trafficking but not lysosomal dysfunction dampens inflammasome activation

Because of their propensity to accumulate lipids, NPC1 mutant cells demonstrate impaired phagosome maturation (Huynh et al., 2008), autophagy (Sarkar et al., 2013), and defective NOD2-mediated microbial clearance (Schwerd et al., 2016). These studies prompted us to investigate whether lysosomal dysfunction in NPC1 mutant cells could account for defective inflammasome activation. To address this, we took advantage of vacuolar-type H^+^-ATPase inhibitor Bafilomycin A1 (Baf A1), which prevents phagosome–lysosome fusion, and chloroquine diphosphate (CQ), an inhibitor of lysosomal acidification. Exposure of LPS-primed cells to either of these during the last 1 h of priming resulted in comparable IL-1β secretion, dismissing reduced inflammasome activation as a secondary outcome of elevated lysosomal cholesterol accumulation (Fig. 1, J and K).

We next performed experiments to determine the role of priming in our assays. Activation of the NLRP3 inflammasome requires two signals. The first signal, which potentiates the NLRP3 inflammasome, is provided upon Toll-like receptor (TLR) ligation and results in the synthesis of pro–IL-1β and up-regulation of NLRP3 expression (Anand et al., 2011a). This first signal (also known as the priming step) is dependent on the transcription factor NF-κB (Bauernfeind et al., 2009; Hornung and Latz, 2010). The second signal, which is more robustly provided by purinergic receptor P2X_7_ agonist ATP, results in the assembly of NLRP3, casp-1, and adaptor protein ASC to form a multiprotein complex (Pelegrin and Surprenant, 2006). One potential explanation for defective inflammasome activation observed in experiments above could be the reduced priming of the NLRP3 inflammasome when sterol trafficking is perturbed. First, we demonstrate that the above results were not solely limited to TLR4 pathway as exposure of U18666a-treated BMDMs to TLR2 agonist Pam3CSK4 followed by ATP stimulation also resulted in abolition of casp-1 activation and IL-1β secretion (Fig. S1, D and E). As expected, these results were entirely dependent on NLRP3 as *Nlrp3^−/−^* iBMDMs displayed blunted casp-1 activation and IL-1β production in cells exposed to inflammasome agonist nigericin (Fig. S1, F and G). Second, there was no reduction in the expression levels of NLRP3 and the secreted levels of the inflammasome-independent cytokine TNF-α, which are both transcriptionally up-regulated, and the adaptor ASC, when homeostatic cholesterol trafficking was abolished by pharmacological treatment in cells primed with either LPS or Pam3 (Figs. 1 D and S1, D, H, I, and J). On the contrary, a modest increase in NLRP3 and secreted levels of TNF-α was observed, likely because of elevation in TLR signaling upon cholesterol accumulation (Yvan-Charvet et al., 2007; Zhu et al., 2008, 2012). However, this did not directly correlate with transcript levels in our study, which were found comparable, indicating an as yet unknown mechanism for posttranscriptional increase (Fig. S1, K–M). Taken together, these data excluded defective lysosomal function and priming as the reason for diminished NLRP3 activation.

### Deficiency in *Npc1* abrogates NLRP3 inflammasome activation

**Figure 2.**
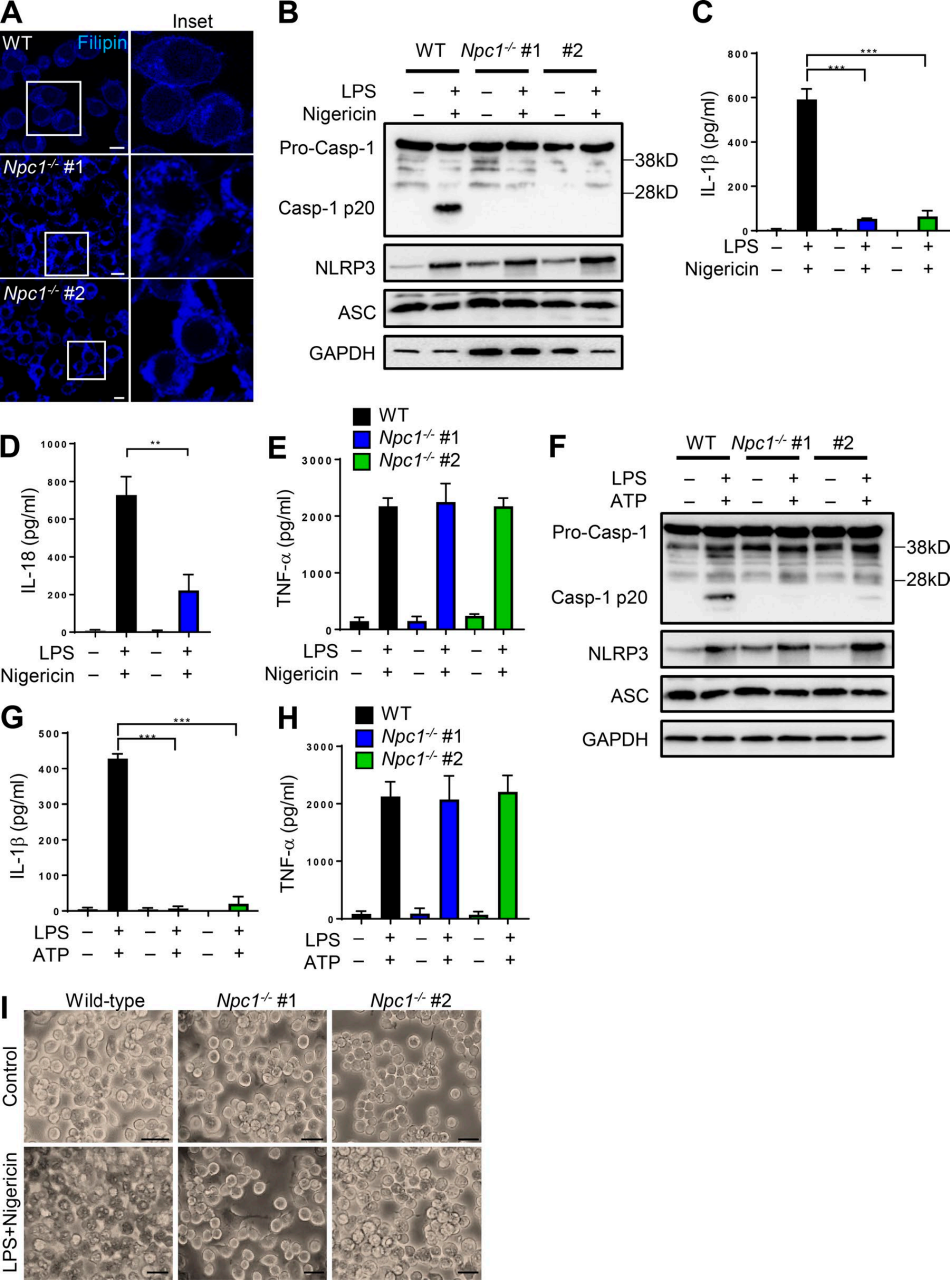
**Deficiency in *Npc1* abrogates NLRP3 inflammasome activation. (A)** Confocal microscopy images of WT and *Npc1^−/−^* iBMDMs stained with filipin. Note that two different clonal cells lines of *Npc1^−/−^* (#1, #2) are shown. Bars, 10 µm. **(B–E)** WT and *Npc1^−/−^* cells were either left untreated or treated with LPS (500 ng/ml; 4 h) and nigericin (20 nM; 45 min). Cell lysates were immunoblotted for antibodies indicated (B), and cell supernatants were analyzed for IL-1β, IL-18, and TNF-α by ELISA (C–E). **(F–H)** WT and *Npc1^−/−^* cells were either left untreated or treated with LPS (500 ng/ml; 4 h) and ATP (5 µM; 45 min). Cell lysates were immunoblotted for antibodies indicated (F), and cell supernatants were analyzed for IL-1β and TNF-α by ELISA (G and H). **(I)** Microscopy images of cells treated as above in B showing characteristic pyroptotic cell death. Bars, 20 µm. Data shown are mean ± SD, and experiments shown are representative of at least three independent experiments. **, P < 0.01; ***, P < 0.001; ****, P < 0.0001, by Student’s *t* test.

Cholesterol egress from lysosomes is directly dependent on sterol transporter NPC1. Next, to validate our results in a genetic model, we generated *Npc1*-deficient iBMDMs using the CRISPR-Cas9 approach (see Materials and methods; Fig. S2). Two independent clonal cell lines were obtained that showed deletion of variable lengths at the *Npc1* locus as validated by Sanger sequencing (Fig. S2, A and B). Reverse transcription–quantitative PCR (RT-qPCR) analysis showed that compared with WT cells, the *Npc1* mRNA levels in both clonal cell lines were significantly reduced (Fig. S2 C). Moreover, in agreement with the known function of NPC1, both mutant cell lines exhibited cholesterol accumulation in distinct punctate structures as revealed by filipin staining (Fig. 2 A). Thus, we concluded that CRISPR-Cas9 targeting resulted in deficiency of NPC1 activity, and therefore, the mutations are genuine loss-of-function alleles.

Exposure of LPS-primed WT cells to inflammasome agonist nigericin resulted in the maturation of 45 kD pro–casp-1 to 20 kD active casp-1. However, activation of casp-1 was ablated in *Npc1*-deficient cells (Fig. 2 B). In agreement, IL-1β and IL-18 release was also significantly abolished in *Npc1*-deficient cells (Fig. 2, C and D). As seen with the pharmacological inhibitor, there was a modest increase in NLRP3 expression in *Npc1-*deficient cells, while secretion of the inflammasome-independent cytokine TNF-α remained unaffected (Fig. 2, B and E). Activation of cells with LPS + ATP reiterated defective casp-1 activation and IL-1β release in *Npc1*-deficient cells, while TNF-α secretion remained unaffected (Fig. 2, F–H). Furthermore, *Npc1*-deficient cells were remarkably protected from cell death (Fig. 2 I). These results thus establish both pharmacological and genetic knockout of *Npc1* as valid approaches to block cholesterol lysosomal egress, and furthermore, they implicate NPC1-dependent cellular sterol trafficking as pivotal for the activation of the NLRP3 inflammasome.

### Lysosomal sterol efflux is not required for the activation of NLRC4 and AIM2 inflammasomes

**Figure 3.**
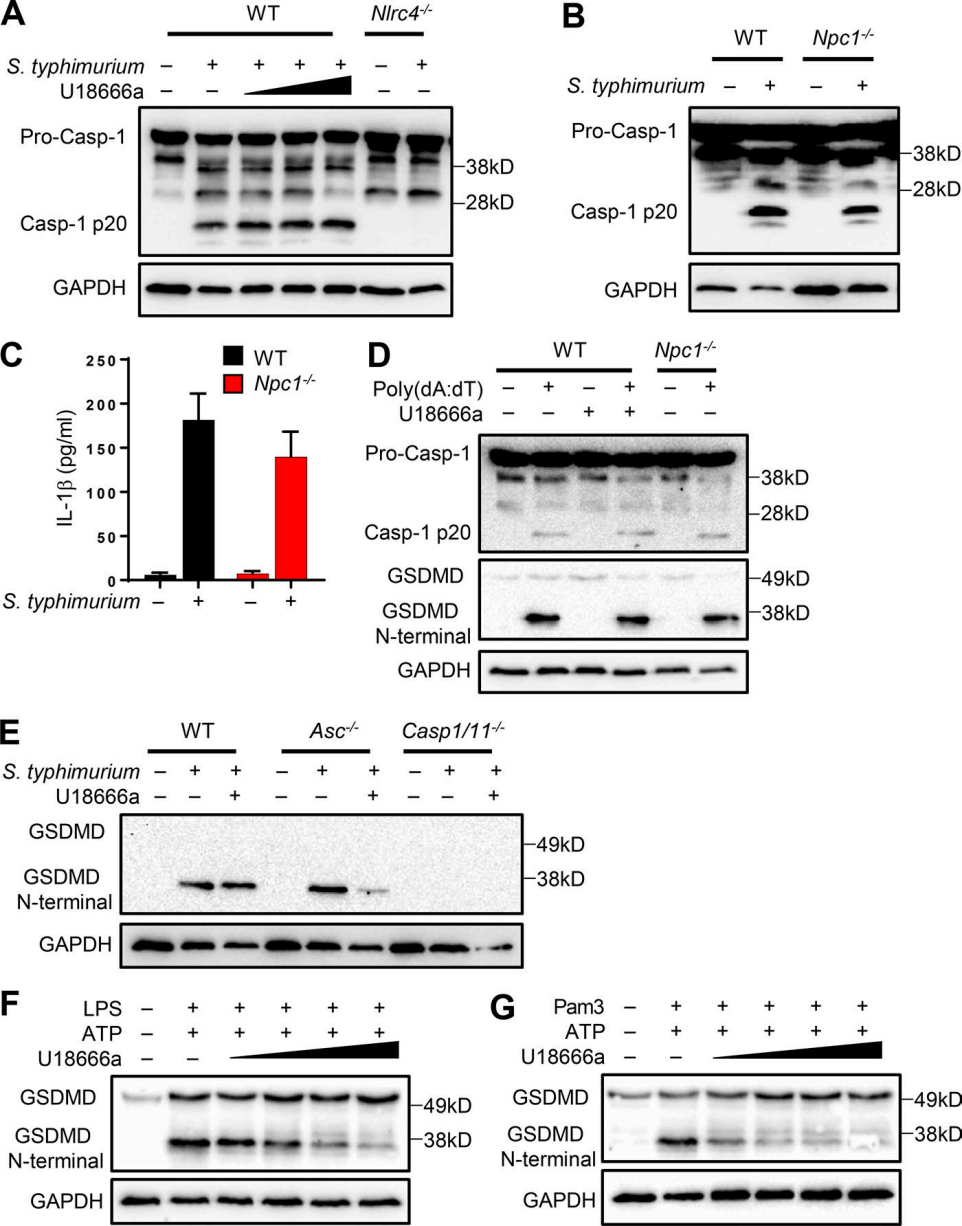
***Npc1* deficiency does not affect the activation of NLRC4 and AIM2 inflammasomes. (A)** WT iBMDMs were incubated with or without the presence of increasing concentrations of U18666a (2, 5, and 10 µg/ml) alongside *Nlrc4*^−/−^ cells and subsequently infected with *S. typhimurium* at an MOI of 2 for ∼4 h. Cell lysates were immunoblotted for casp-1 and GAPDH. **(B)** WT and *Npc1^−/−^* cells were treated with *S. typhimurium* for 4 h and immunoblotted as in A. **(C)** Cell supernatants were analyzed for IL-1β. **(D)** WT cells either treated or not with U18666a (5 µg/ml) and *Npc1^−/−^* cells were transfected with poly(dA:dT) for 4 h before cell lysates were immunoblotted for the antibodies indicated. **(E)** WT, *Asc*^−/−^, and *caspase 1/11*^−/−^ cells were exposed or not to U18666a before infection with *S. typhimurium* as above. Cell lysates were immunoblotted for GSDMD and GAPDH. **(F and G)** BMDMs were treated with either LPS (500 ng/ml; 4 h) or Pam3 (500 ng/ml; 4 h) in the presence of increasing concentrations of U18666a (1, 2, 5, and 10 µg/ml) followed by ATP (5 mM; 45 min). Cell lysates were immunoblotted for GSDMD and GAPDH.

A diverse array of signals is known to activate the NLRP3 inflammasome. In contrast, ligands that activate the NLRC4 and AIM2 inflammasomes are well defined. The NLRC4 inflammasome is triggered by the recognition of cytosolic flagellin and components of the bacterial secretion system by upstream NAIP family members (Amer et al., 2006; Kofoed and Vance, 2011; Zhao et al., 2011). AIM2 inflammasome, in contrast, is activated by recognition of double-stranded DNA of ∼80 bp in length (Jin et al., 2012). We next tested whether the requirement for homeostatic cholesterol transport is specific to the activation of the NLRP3 inflammasome. First, we tested whether lysosomal sterol accumulation affects NLRC4 inflammasome activation by infecting macrophages with *Salmonella typhimurium.* Unlike NLRP3, casp-1 activation by the NLRC4 inflammasome remained unaffected in cells where NPC1 was pharmacologically blocked (Fig. 3 A). In agreement, *Npc1^−/−^* cells also demonstrated casp-1 activation and IL-1β secretion comparable with that observed in infected WT cells (Fig. 3, B and C). We next evaluated whether the DNA sensing AIM2 inflammasome is affected by sterol lysosomal compartmentalization. To evaluate this, WT cells (in the presence or absence of U18666a) and *Npc1^−/−^* cells were transfected with poly(dA:dT). Western blotting revealed no impact on casp-1 activation upon poly(dA:dT) transfection when lysosomal egress of cholesterol was blocked (Fig. 3 D). These results demonstrate that sterol lysosomal compartmentalization uniquely affects the NLRP3 inflammasome.

### Cholesterol compartmentalization dampens GSDMD-mediated ASC-independent pyroptosis following NLRC4 inflammasome activation

Activation of the inflammatory caspases (including casp-1) results in the cleavage of GSDMD to remove the autoinhibitory C-terminal fragment. The resulting active N-terminal cleavage product oligomerizes to form membrane pores to trigger pyroptosis (Kayagaki et al., 2015; Shi et al., 2015; Ding et al., 2016). Previous research demonstrated that induction of pyroptosis, as demonstrated by LDH release during NLRC4 inflammasome activation by *S. typhimurium*, is ASC-independent but casp-1–dependent. Surprisingly, NLRC4-dependent pyroptosis progressed in the absence of casp-1 autoproteolysis (Broz et al., 2010). We therefore took advantage of these studies to assess whether sterol lysosomal efflux influenced the induction of ASC-independent pyroptosis. As observed above, blockade of cholesterol transport in WT cells did not inhibit casp-1 activation by *S. typhimurium* infection (Fig. 3 A). Accordingly, cleavage of GSDMD was also similar when NPC1 was pharmacologically blocked in WT cells (Fig. 3 E). In agreement with previous research (Broz et al., 2010), pyroptosis, and thus GSDMD N-terminal fragment, was not observed in *casp-1/11^−/−^* cells but was cleaved in *Asc^−/−^* cells comparably with WT cells (Fig. 3 E). Surprisingly, *Asc^−/−^* cells treated with U18666a exhibited reduced GSDMD cleavage, suggesting a role for homeostatic cholesterol transport in pyroptosis only in the absence of ASC (Fig. 3 E). By contrast, cholesterol accumulation had no effect on GSDMD cleavage either in U18666a-treated or in *Npc1^−/−^* cells following AIM2 inflammasome activation by poly(dA:dT) transfection (Fig. 3 D). Conversely, GSDMD cleavage was reduced following NLRP3 inflammasome activation with both LPS + ATP and Pam3 + ATP, mirroring the effects seen on casp-1 activation (Fig. 3, F and G). These data thus suggest that cholesterol lysosomal egress is required for ASC-independent pyroptosis following NLRC4 activation. However, pyroptosis following activation of the AIM2 inflammasome is not affected.

### Cholesterol supplementation restores inflammasome activation

Variation in the levels of cholesterol and other lipids within the cell are sensed by master transcriptional regulators SREBP1 and SREBP2, which, respectively regulate fatty acid and cholesterol biosynthesis (Horton et al., 2002). A feedback mechanism tightly regulates cholesterol de novo biosynthesis to maintain homeostatic levels. Cholesterol depletion triggers ER resident sterol cargo protein SCAP to escort SREBP2 to the Golgi, where the precursor is cleaved to activate cholesterogenic genes. By contrast, in cholesterol-replete cells, conformational change in SCAP promotes insulin-induced gene–mediated retention of SREBP2 in the ER.

Low ER cholesterol is an established trigger for SREBP2 activation. Although the movement of cholesterol from the NPC1 compartment to the PM and to the ER is defective in cells lacking functional NPC1 (Cruz et al., 2000; Sugii et al., 2003), the total cholesterol levels were not considerably elevated when NPC1 was pharmacologically blocked (Fig. 1 A). To investigate this further, we quantified SREBP2 targets hydroxymethylglutaryl (HMG)-CoA synthase (*Hmgcs1*) and HMG-CoA reductase (*Hmgcr*) in LPS-primed cells, and the transcript levels were significantly down-regulated in *Npc1^−/−^* cells (Fig. S2, D–F). Intriguingly, the expression of *Srebp2* was also reduced in *Npc1*-deficient cells, thereby reconfirming previous research showing that SREBP2 autoregulates its own mRNA expression (Sato et al., 1996). The decreased expression of SREBP2-dependent genes likely also results in reduced nuclear localization of the mature protein as correlation has been observed in other studies (Repa et al., 2000; Im et al., 2011). The levels of *Srebp2* and the target gene *Hmgcs1* were elevated in control *Npc1^−/−^* cells (Fig. S4 A).

SREBP-dependent lipogenesis is regulated by mTORC1, a master regulator of cellular metabolism (Porstmann et al., 2008; Peterson et al., 2011). By evaluating the phosphorylation of ribosomal protein p70 S6 kinase 1 (S6K1), a direct mTOR substrate, we investigated the upstream pathway that modulates SREBP2 in our study. *Npc1^−/−^*- and U18666a-exposed cells displayed reduced phosphorylation of S6K1 and the upstream mTOR regulator serine/threonine protein kinase AKT (protein kinase B) in cells exposed to LPS in a time-course experiment (Fig. S3). The decrease in the activation of AKT–mTOR pathway is likely a result of disorganized lipid raft microdomains in the PM of *Npc1^−/−^* cells (Simons and Toomre, 2000; Vainio et al., 2005). Accordingly, *Npc1^−/−^* cells exhibited reduced PM cholesterol levels as measured by its susceptibility to cholesterol oxidase (Fig. S4 B). Indeed, reduction in PM cholesterol in WT cells by methyl-β-cyclodextrin (MCD) treatment resulted in reduced mTOR activity (unpublished data). Furthermore, inhibition of mTOR by selective inhibitor Torin1 significantly decreased the expression of *Srebp2* and the target genes *Hmgcs1* and *Hmgcr* (Fig. S2, G–I). The regulation of SREBP2 by mTOR is well established and involves the phosphatidic acid phosphatase lipin-1, which functions at several steps to control SREBP activity (Peterson et al., 2011). These results demonstrate reduced mTOR- and SREBP2-dependent lipogenesis in *Npc1^−/−^* cells following LPS stimulation.

**Figure 4.**
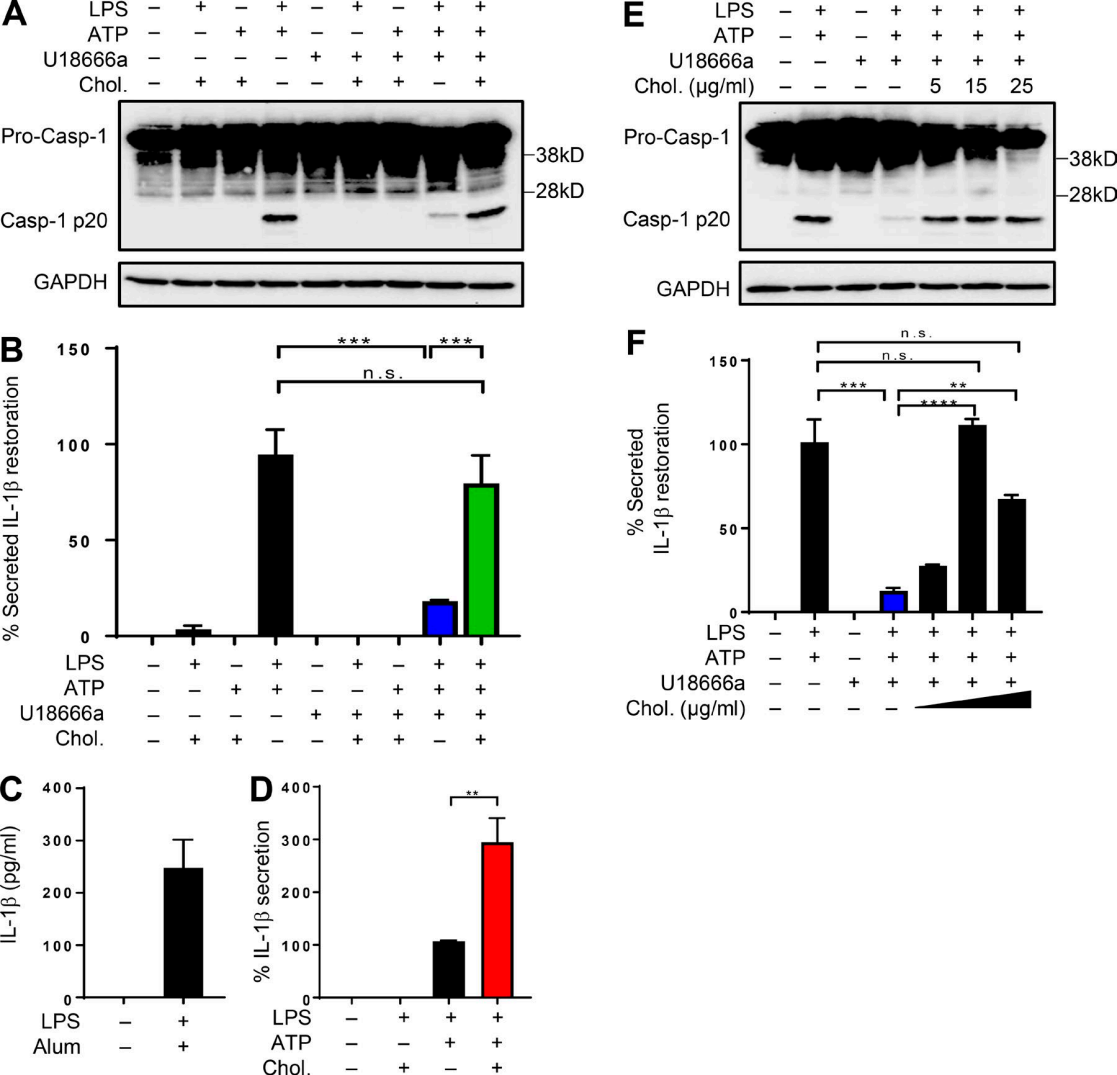
**Cholesterol supplementation restores inflammasome activation in cells defective in NPC1 function. (A)** BMDMs were either left untreated or exposed to LPS and cholesterol–MCD complexes (chol), cholesterol and ATP, or LPS and ATP in the presence or absence of 5 µg/ml U18666a. Where added, cells were incubated with 15 µg/ml cholesterol for 1 h before ATP treatment. Samples were immunoblotted with casp-1, and GAPDH was used as a loading control. **(B)** Cell supernatants were analyzed for secreted IL-1β by ELISA. Bar graph shows percent IL-1β restoration when cholesterol–MCD was added. **(C)** IL-1β levels in LPS-primed BMDMs grown in complete DMEM and exposed to alum (1 mg/ml). **(D)** IL-1β levels in *Npc1^−/−^* cells either left untreated or treated with LPS and cholesterol–MCD for 1 h followed by ATP. **(E)** BMDMs were either left untreated or exposed to LPS and ATP in the presence or absence of 5 µg/ml U18666a. Where added, cells were incubated with indicated concentrations of cholesterol–MCD for 1 h before ATP treatment. Samples were immunoblotted with the indicated antibodies. GAPDH was used as a loading control. **(F)** Cell supernatants from above were analyzed for secreted IL-1β by ELISA. Bar graph shows percent IL-1β restoration when cholesterol–MCD was added. Data shown are mean ± SD, and experiments shown are representative of at least three independent experiments. **, P < 0.01; ***, P < 0.001; ****, P < 0.0001, by Student’s *t* test.

Since our observations above suggested overall reduction in cholesterol in subcellular organelles, we reasoned that supplementation with exogenous cholesterol should restore inflammasome activity. To conduct these experiments, MCD-solubilized cholesterol was added to LPS-primed cells cultured in serum-free medium 1 h before ATP treatment to assess only the effects on the second signal of inflammasome activation. Remarkably, supplementation with soluble cholesterol restored inflammasome activation in cells where lysosomal egress of cholesterol was pharmacologically blocked, resulting in a distinct increase in both casp-1 cleavage and IL-1β secretion (Fig. 4, A and B). The addition of cholesterol–MCD complex to LPS-primed cells in the absence of ATP was insufficient to activate the inflammasome, while exposure to alum resulted in abundant IL-1β secretion, suggesting the crystal-independent nature of cholesterol–MCD-induced NLRP3 inflammasome (Fig. 4, B and C). Similarly, cholesterol–MCD by itself was insufficient at providing “signal 1” in the absence of LPS (Fig. 4, A and B). *Npc1^−/−^* cells supplemented with exogenous cholesterol–MCD reiterated elevated IL-1β secretion (Fig. 4 D). Also, this response appeared to be concentration dependent, with the optimal concentration of cholesterol peaking at ∼15 µg/ml (Fig. 4, E and F). Collectively, these results demonstrate that replenishment with soluble cholesterol in cells with nonfunctional NPC1 restores casp-1 activation and IL-1β production.

### Cholesterol depletion in the ER membranes abrogates casp-1 activation and IL-1β secretion

**Figure 5.**
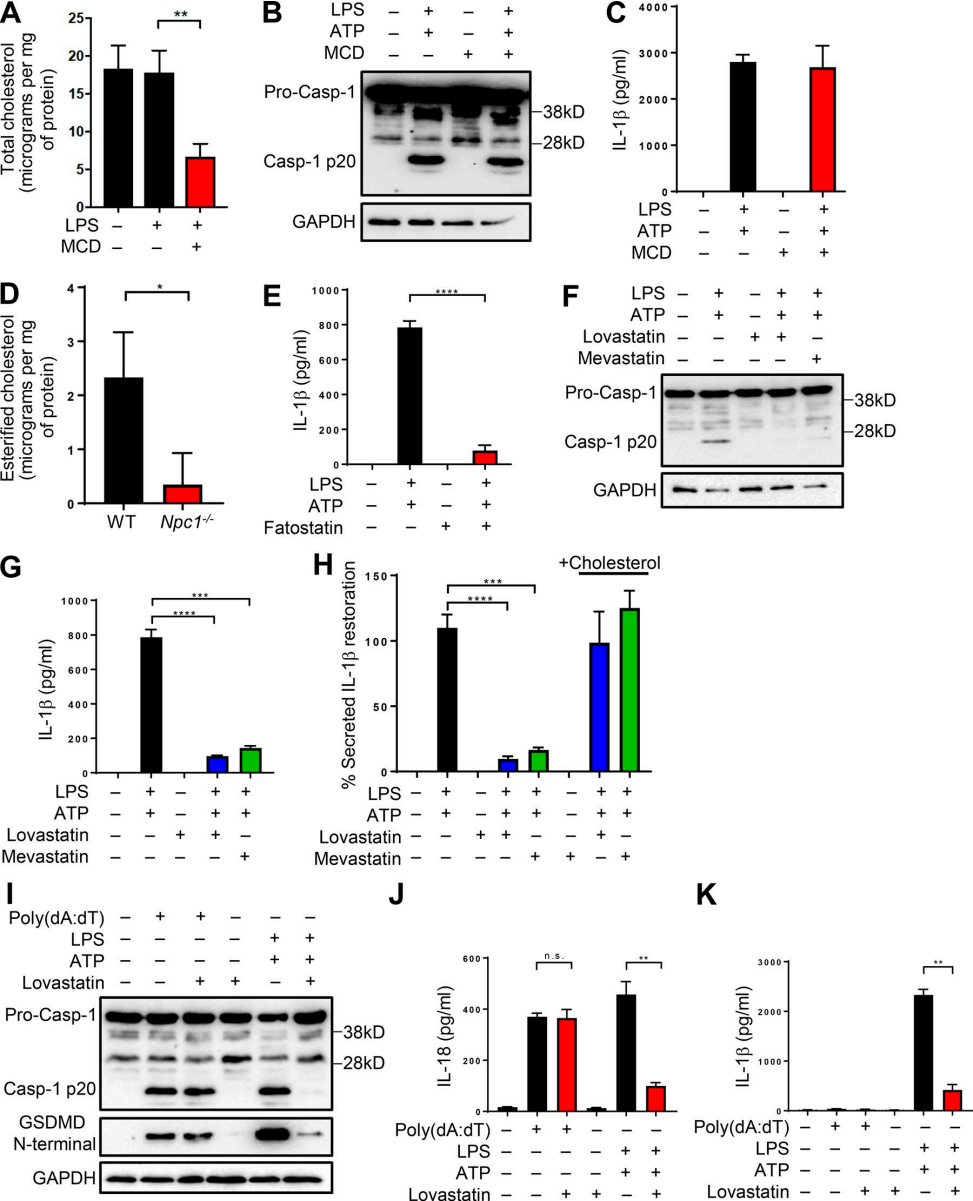
**Cholesterol depletion in the ER membranes abrogates casp-1 activation and IL-1β secretion. (A)** Total cholesterol levels in control or LPS treated BMDMs in the presence or absence of MCD. **(B and C)** BMDMs were treated as in A followed by the addition of ATP. Cell lysates were immunoblotted for casp-1 (B), and supernatants were analyzed for IL-1β (C). **(D)** WT and *Npc1^−/−^* cells were treated with LPS (500 ng/ml; 4 h) before measurement of esterified cholesterol. **(E and G)** IL-1β levels in LPS-primed BMDMs exposed to fatostatin (E), lovastatin, or mevastatin (G) for 1 h before the addition of ATP. **(F)** Casp-1 immunoblotting of cell lysates treated as described above and indicated. **(H)** Secreted IL-1β levels upon addition of cholesterol–MCD for 1 h before ATP stimulation from BMDM treated as above in G. **(I)** BMDMs were either treated with LPS + ATP or transfected with poly(dA:dT) (1 µg/ml; 4 h) in the presence or absence of lovastatin (40 µM) during the final 1 h. Cell lysates were immunoblotted for the antibodies indicated. **(J and K)** Cells were treated as in I, and cell supernatants were analyzed for IL-18 (J) and IL-1β (K). Data shown are mean ± SD, and experiments shown are representative of at least three independent experiments. *, P < 0.05; **, P < 0.01; ***, P < 0.001; ****, P < 0.0001, by Student’s *t* test.

We next investigated the subcellular site to which the cholesterol must localize for optimal inflammasome activity. To address this, we first focused on the PM, which is the largest pool of cholesterol in the cell, where it makes up 50–60% of total lipids (Ridsdale et al., 2006). To measure the contribution of PM, we took advantage of a routinely used cholesterol extraction agent, MCD, to deplete PM cholesterol from cells cultured in lipoprotein-deficient serum. We recognized from our experiments above that changes to the PM cholesterol affect AKT–mTOR signaling. Therefore, to eliminate the involvement of other pathways and to specifically address the role of PM cholesterol in the second step of inflammasome activation, we exposed LPS-primed BMDMs to MCD only for 30 min before ATP stimulation. We reasoned that restricted exposure to MCD would affect only the PM cholesterol while retaining the architecture of other cellular cholesterol pools (Ridsdale et al., 2006). As expected, exposure to MCD resulted in reduction in total cholesterol levels by ∼60% (Fig. 5 A). However, subsequent treatment with ATP did not affect casp-1 activation or IL-1β release (Fig. 5, B and C), excluding any role for PM cholesterol in inflammasome activation.

The transport of cholesterol from the NPC1 compartment to the ER is blunted in cells lacking functional NPC1 (Cruz et al., 2000; Sugii et al., 2003). In agreement, *Npc1*^−/−^ cells exhibited diminished levels of esterified cholesterol (Fig. 5 D). Therefore, we next examined the role of cholesterol in the ER membranes, which are cholesterol poor, with only 3–6% of total lipids. To specifically deplete cholesterol in the ER, we took advantage of the fact that cells grown in lipoprotein-deficient serum depend entirely on endogenous cholesterol synthesis to support growth (Ridsdale et al., 2006). We therefore exposed LPS-primed cells to statins, specific inhibitors of cholesterol biosynthetic pathway, to cause acute cholesterol depletion of the ER pool. Statin treatment initially decreases cholesterol specifically in the ER membranes; therefore, we restricted statin treatment to only 1 h before ATP stimulation. In agreement with its function, exposure of control cells to fatostatin resulted in reduced expression of SREBP2 and the target genes (Fig. S4 C). Inflammasome activation in cells exposed to fatostatin resulted in significantly reduced IL-1β secretion (Fig. 5 E). Similarly, treatment with lovastatin and mevastatin, which target HMG-CoA reductase, the rate limiting enzyme of cholesterol synthesis, also resulted in reduced casp-1 cleavage and IL-1β secretion upon NLRP3 activation (Fig. 5, F and G). Conversely, addition of either cholesterol–MCD or mevalonate, the direct product of HMG-CoA reductase, to the media of statin-exposed cells restored IL-1β secretion (Fig. 5 H and Fig. S4 D). Statin treatment, however, affected only the NLRP3 inflammasome. Exposure of cells grown in lipoprotein-deficient media to lovastatin during the last 1 h of AIM2 inflammasome activation by poly(dA:dT) transfection did not affect casp-1 activation (Fig. 5 I). Consequently, secretion of IL-18 was also comparable regardless of the presence of lovastatin during poly(dA:dT) transfection (Fig. 5 J). As expected, the secretion of IL-18 and IL-1β was significantly diminished during LPS and ATP stimulation (Fig. 5, J and K). These observations unequivocally demonstrate that ER cholesterol is required for the NLRP3 inflammasome activation.

### ER cholesterol depletion blunts ASC-dependent inflammasome assembly

**Figure 6.**
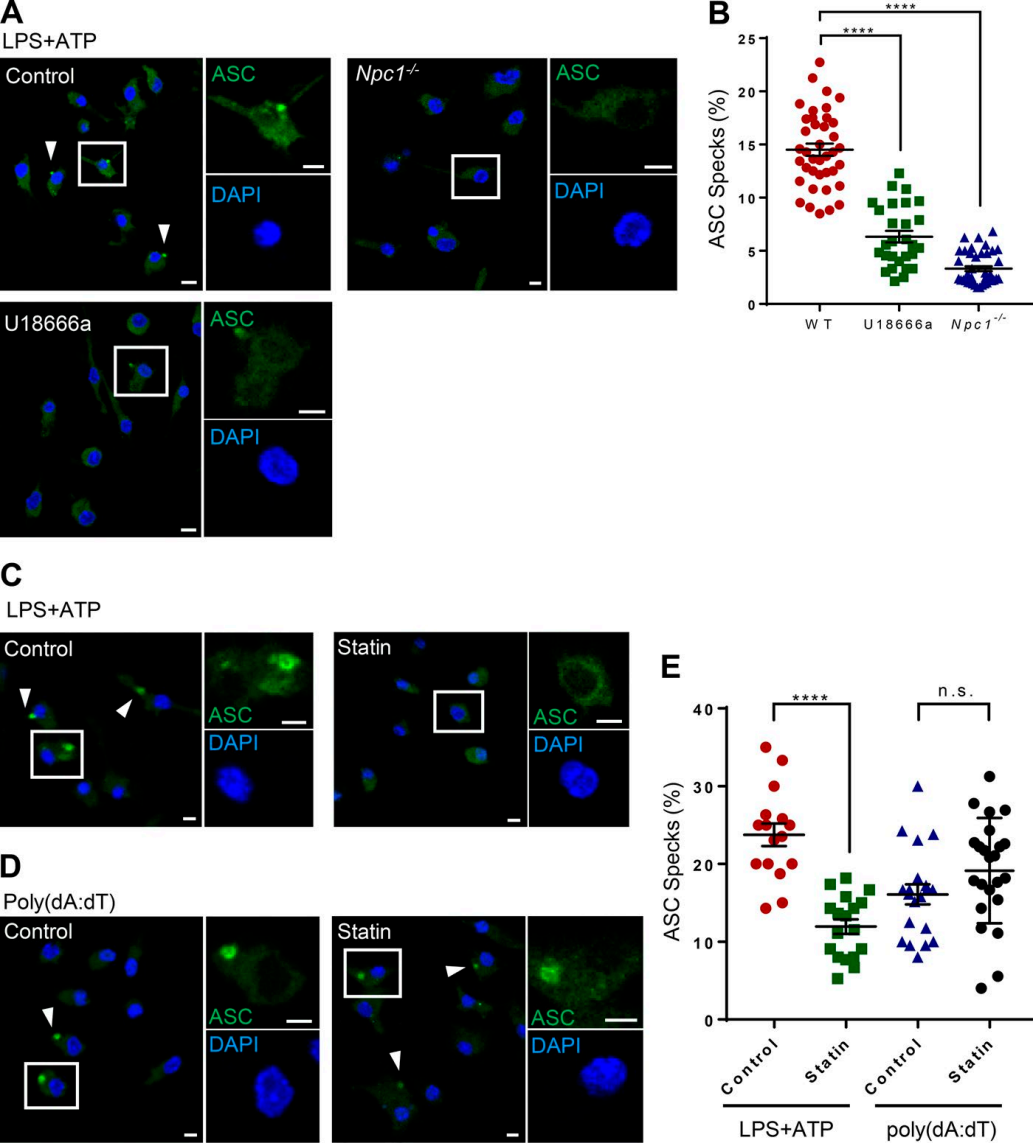
**ER cholesterol depletion blunts ASC-dependent inflammasome assembly. (A)** WT (control), U18666a-treated, and *Npc1^−/−^* cells were exposed to LPS + ATP followed by labeling with anti-ASC antibody and DAPI staining. **(B)** Quantitative analysis of percentage of cells with ASC specks in samples treated as above. Each dot represents an individual field with at least *n* = 40 cells. **(C and D)** LPS-primed BMDMs exposed to ATP (C) or poly(dA:dT)-transfected BMDMs (D) were exposed or not to lovastatin (40 µM; 1 h) followed by labeling with anti-ASC antibody and DAPI staining. **(E)** Quantitative analysis of percentage of cells with ASC specks in samples treated as above. Each dot represents an individual field with at least *n* = 30 cells. Data shown are mean ± SEM, and experiments shown are representative of at least three independent experiments. Arrowheads show ASC specks. Bars, 5 µm. ****, P < 0.0001, by Student’s *t* test.

Activation of inflammasome and IL-1β secretion is critically dependent on the assembly of the supramolecular complex containing NLRP3, ASC, and casp-1. The assembly of this complex is quite elaborate and requires the release to the cytoplasm of mitochondria-associated ER membrane (MAM)-localized NLRP3, which in the presence of the activating stimuli, nucleates the ASC filament, believed to be a major determinant for inflammasome assembly and the downstream cellular functions of inflammasomes. ASC, a bipartite protein composed of a PYD and a CARD domain, acts as an adaptor protein linking the PYD-containing NLRP3 to the CARD-containing casp-1 through homotypic interactions. Upon receptor activation, ASC oligomerizes to form the so-called ASC speck, a macromolecular complex that is ∼1–2 µm in diameter. Under resting conditions, ASC is present diffusely throughout the cytoplasm. Stimulation of the NLRP3 inflammasome with LPS + ATP was sufficient to assemble the inflammasome, resulting in ASC speck formation in control WT cells (Fig. 6 A). However, ASC speck formation was significantly abolished both in *Npc1^−/−^* cells and in cells where NPC1 was pharmacologically blocked by U18666a (Fig. 6, A and B). We next independently verified whether ER cholesterol depletion by statin treatment could also blunt the assembly of the NLRP3 inflammasome. As before, we grew BMDMs in lipoprotein-deficient media and depleted ER cholesterol by exposing LPS-primed cells to lovastatin for 1 h before ATP stimulation. In response to NLRP3 agonist ATP, control cells revealed expected levels of ASC speck formation. However, the percentage of cells showing ASC specks was significantly reduced in samples treated with lovastatin (Fig. 6, C and E). Similarly, control cells exposed to AIM2 agonist poly(dA:dT) demonstrated ASC speck formation. However, in contrast to the NLRP3 inflammasome, ER cholesterol depletion by exposure to lovastatin during the last 1 h of poly(dA:dT) transfection did not influence the assembly of the AIM2 inflammasome (Fig. 6, D and E). These results thus suggest that NLRP3 complex formation is distinctly regulated by the ER cholesterol pool.

### NLRP3 recruitment to ASC is diminished in *Npc1-*deficient cells

**Figure 7.**
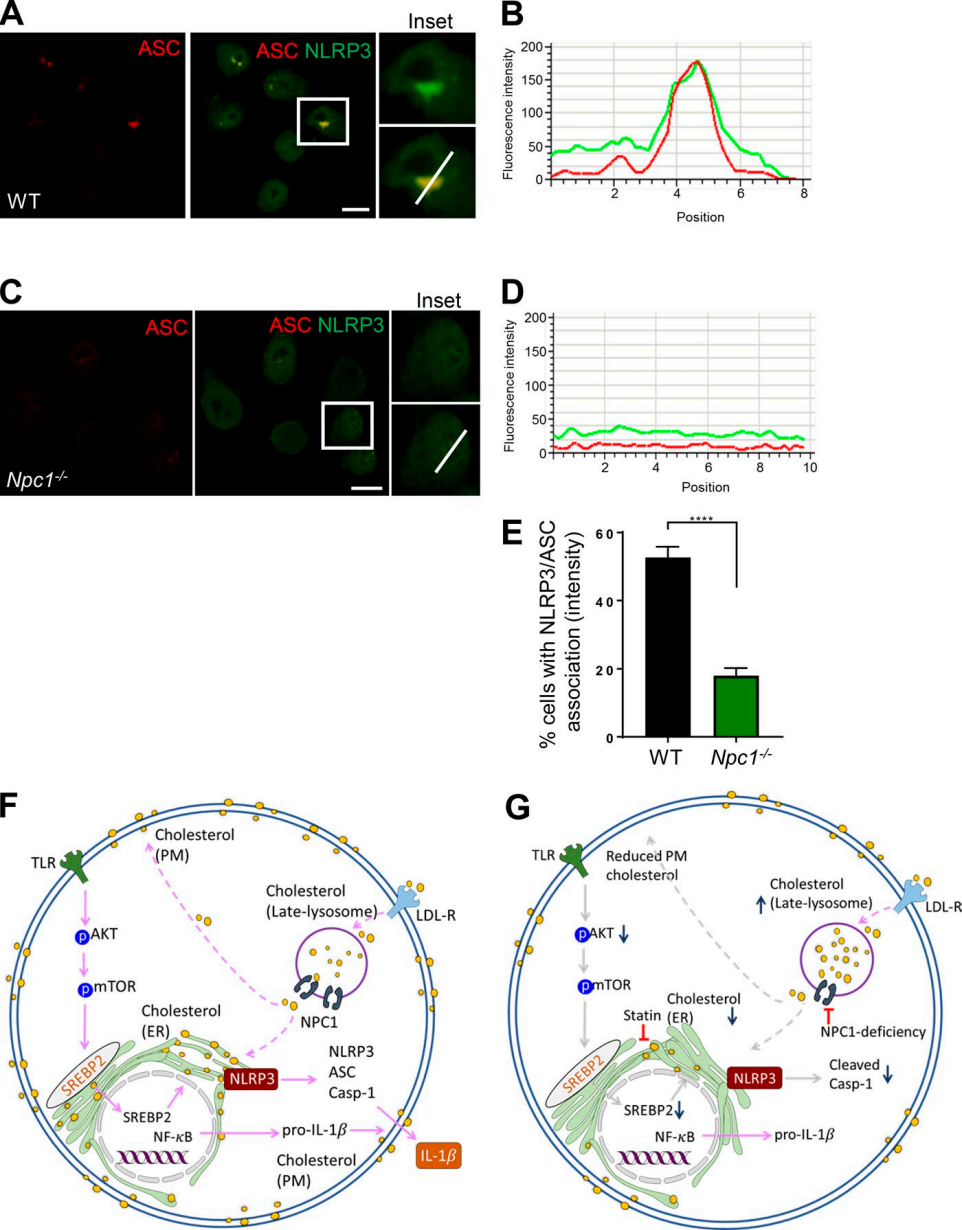
**NLRP3 recruitment to ASC is diminished in *Npc1^−/−^* cells. (A and C)** WT and *Npc1^−/−^* cells were exposed to LPS + ATP and subsequently fixed and labeled for NLRP3 and ASC with secondary antibodies conjugated to FITC and Alexa Fluor 546, respectively. Inset shows zoom of indicated regio­n on the middle panel. Bars, 10 µm. **(B and D)** Profile of fluorescence intensity along the indicated white bar in inset in A and C, respectively. **(E)** Quantitative analysis showing the percentage of cells that show NLRP3 and ASC association. At least 10 random fields were selected each with *n* = 15 cells. Data shown are mean ± SD, and experiments shown are representative of at least three independent experiments. ****, P < 0.0001, by Student’s *t* test. **(F and G)** Schematic model describing the identified mechanism in the activation of the NLRP3 inflammasome. **(F)** Homeostatic cellular cholesterol trafficking to the ER is required for optimal inflammasome activation. Exogenously obtained LDL bound to LDL-R is endocytosed at the PM. Cholesterol efflux from the late endosome–lysosome compartment to other cellular sites such as the PM and the ER is dependent on NPC1. **(G)** Blockade of the cholesterol transporter NPC1 leads to lysosomal cholesterol accumulation and a subsequent decrease in cholesterol pool in both the ER and the PM. This in turn leads to decreased phosphorylation of AKT and mTOR following TLR ligation, causing a reduction in SREBP2-dependent lipogenesis. Additionally, perturbation in the ER cholesterol levels leads to decreased association of NLRP3 and ASC, resulting in reduced active casp-1 levels and IL-1β secretion. Furthermore, inhibition of ER cholesterol levels by statins similarly blunts NLRP3 inflammasome activation. LDL-R, low-density lipoprotein receptor; mTOR, mammalian target of rapamycin; SREBP2, sterol regulatory element binding protein 2.

Biochemical evidence suggests that NLRP3 is mainly localized on MAMs, thus positioning NLRP3 in close proximity to mitochondria-released effectors, many of which are known to activate the NLRP3 inflammasome (Nakahira et al., 2011; Zhou et al., 2011; Iyer et al., 2013; Lupfer et al., 2013, 2014; Wang et al., 2013; Gurung et al., 2015). On the contrary, the majority of the ASC is found in the cytoplasmic fraction before inflammasome activation, although a minor portion of ASC is found associated with the ER fraction (Zhou et al., 2011). However, the fully active inflammasome complex is mostly cytoplasmic, suggesting the requirement of additional steps in the activation of the inflammasome (Zhou et al., 2011; Wang et al., 2013). Intriguingly, the described distribution of the activating receptor is specific to the NLRP3 inflammasome, while localization of non-NLRP3 inflammasomes remains less characterized. We next tested the localization of NLRP3. In agreement with previous studies (Zhou et al., 2011; Zhang et al., 2017), control LPS-primed cells exhibited a fraction of NLRP3 close to MAMs as revealed by double labeling with the ER marker calreticulin (Fig. S5 A). Upon subsequent exposure to ATP, NLRP3 was found strongly associated to ASC in WT cells (Fig. 7, A and B). However, this was significantly diminished in *Npc1^−/−^* cells, where NLRP3 and ASC were both found diffusely localized throughout the cell (Fig. 7, C and D). Quantitative analysis revealed a signification reduction in NLRP3 association with ASC in *Npc1*-deficient cells (Fig. 7 E). As expected, the assembled inflammasome did not show significant association with ER, while mitochondria were found in close proximity (Fig. S5 B). However, in agreement with another study (Zhou et al., 2011), ASC speck was found associated with mitochondria and ER in a fraction of cells. Our study thus suggests that ER cholesterol is pivotal for recruitment of NLRP3 to ASC and thus full activation of the NLRP3 inflammasome. Overall, our data suggest that cholesterol acts as a rheostat to modulate NLRP3 activity, and thus these studies may be useful to develop novel therapeutics to treat NLRP3-related disorders.

## Discussion

Our study demonstrates that homeostatic sterol trafficking and distribution provide an appropriate intracellular milieu for the activation of the NLRP3 inflammasome. Pharmacological or genetic blockade of NPC1 blunted NLRP3 inflammasome activation, resulting in reduced casp-1 activation and IL-1β secretion in response to NLRP3 agonists. Remarkably, cellular cholesterol trafficking uniquely affects the NLRP3 inflammasome, while activation of the NLRC4 and AIM2 inflammasomes remained unimpeded. Further mechanistic research suggested that cholesterol trafficking to the ER licenses the NLRP3 inflammasome activation. In agreement, acute depletion of the ER cholesterol pool by statin treatment abrogated the assembly and activation of the NLRP3 inflammasome.

A previous study associated inflammasome activation to dietary cholesterol (Progatzky et al., 2014). These authors observed an increase in the accumulation of myeloid cells in the intestine of zebrafish that were fed a high-cholesterol diet, which was found to be dependent on cholesterol uptake by PM–localized NPC1-like 1 (NPC1L1). Inhibitors of both NPC1L1 and inflammasome components reduced the accumulation of myeloid cells. This study thus provided evidence that cholesterol uptake by NPC1L1 is able to activate the inflammasome (i.e., provide “signal 2”). However, inflammasome activation by cholesterol uptake was restricted to intestinal epithelial cells, with no role identified for hematopoietic cells (Progatzky et al., 2014). In agreement, NPC1L1 is expressed predominately in the gastrointestinal tract, whereas lysosomal NPC1 is more abundantly expressed in a wide variety of cells and tissues (Altmann et al., 2004). Our study signifying the role of macrophage sterol trafficking in NLRP3 activation thus unravels distinct mechanisms of NPC1 and NPC1L1 in modulating inflammasome in discrete cell types.

Previous studies have highlighted a role for cholesterol in NLRP3 inflammasome activation during atherosclerosis (Duewell et al., 2010; Rajamäki et al., 2010; Freigang et al., 2011; Sheedy et al., 2013). Mice fed an atherogenic diet displayed cholesterol crystal formation as early as 2 wk in atherosclerotic lesions, which correlated with macrophage recruitment (Duewell et al., 2010). The inflammasome activation in the above research was dependent on macrophage lysosomal rupture and subsequent cathepsin B release into the cytoplasm following accumulation of cholesterol crystals within the degradative organelle (Hornung et al., 2008). Whether cellular cholesterol trafficking directly impacts inflammasome activation remained unknown. Our data demonstrate that blockade of cholesterol trafficking through NPC1 dampened the NLRP3 inflammasome, which could be restored by addition of exogenous cholesterol. These findings are in agreement with a recently published study in which exogenous cholesterol was shown to activate the AIM2 inflammasome (Dang et al., 2017). However, in their study, cells were exposed to cholesterol–MCD for 8 h, which eventually resulted in mitochondrial dysfunction and increased cytosolic mitochondrial DNA content. Thus, inflammasome activation in their research was dependent on DNA sensor AIM2. However, we exposed macrophages for only 1 h with cholesterol–MCD followed by a canonical NLRP3 stimulus, ATP, thereby implicating a role for NLRP3 inflammasome in our study. Together, these studies demonstrate that cholesterol function in distinct organelles modulates different inflammasomes.

In cells lacking functional NPC1, the movement of cholesterol from the NPC1 compartment to the ER and to the PM is defective (Sugii et al., 2003; Wojtanik and Liscum, 2003). In agreement, *Npc1^−/−^* cells exhibited both reduced levels of esterified cholesterol and decreased sensitivity to cholesterol oxidase. Thus, *Npc1* deficiency alters PM order and composition, thereby affecting discrete cholesterol-dependent signaling networks localized in lipid rafts (Simons and Ehehalt, 2002). This is most likely the reason for both the reduced activation of the AKT–mTOR–SREBP2 axis and a modest increase in *Npc1^−/−^* cells of the NLRP3 expression, the latter agreeing with elevated TLR signaling when cellular sterol efflux is blocked (Zhu et al., 2008). However, it is difficult to reconcile these data at the moment, considering that contrasting responses were observed with signal emanating from the same TLR4 receptor. However, our data would imply an unidentified posttranscriptional mechanism for enhanced TLR responses upon cholesterol accumulation.

We found a key role for the ER cholesterol pool in inflammasome activation, while deviation in the PM cholesterol levels did not affect casp-1 activation and IL-1β secretion. Our model suggesting a significant role for ER cholesterol agrees with previous studies (Fig. 7). Inhibition of SREBP processing by 25-hydroxy cholesterol results in reduced IL-1β production (Reboldi et al., 2014). Likewise, BMDMs lacking SCAP, which chaperones SREBP2 to the Golgi for processing, exhibit reduced casp-1 activation and IL-1β expression (Reboldi et al., 2014). Under resting conditions, NLRP3 is mainly localized on MAMs (Zhou et al., 2011), which are cholesterol poor and sensitive to changes in cholesterol levels. NLRP3 release from MAMs is necessary to oligomerize with cytoplasmic ASC when a second signal is available (Zhou et al., 2011; Wang et al., 2013; Zhang et al., 2017). ER is known to influence innate immune signaling, but the underlying mechanisms remain unclear (Toulmay and Prinz, 2011; Misawa et al., 2017). Together with our data, this raises an intriguing possibility that ER lipid composition might play a direct or indirect function in modulating immune responses, including the activation of inflammasome.

We observed a significantly reduced association of NLRP3 and cytoplasmic ASC in *Npc1^−/−^* cells, suggesting that cholesterol likely provides fluidity to the ER membranes to allow NLRP3 release and subsequent assembly of an active inflammasome. However, at this time, it is difficult to rule out the additional roles of ER cholesterol upstream of the inflammasome assembly formation and NLRP3 activation. For example, ER cholesterol may be required to provide the necessary conformation to NLRP3 to sense the activating effectors. Similarly, ER cholesterol might also enable NLRP3 association with ASC by allowing the extension of the ER tubules before the dissociation of the complex from the ER. In agreement with this, a fraction of cells displayed ASC specks that exhibited association with calreticulin (not depicted). Furthermore, in the latter scenario, cholesterol might also have a direct role in inflammasome assembly. It will be interesting to dissect the precise roles of ER cholesterol in inflammasome assembly and activation in future studies. Overall, our study reveals an essential requirement for sterol trafficking to the ER for the activation of the NLRP3 inflammasome. The results presented in our study may provide clues to develop novel treatment avenues for diseases where dysregulated lipid metabolism is the underlying factor.

## Materials and methods

### Ethics statement

Experiments were performed in accordance with the Animals (Scientific Procedures) Act 1986 and were approved by the Imperial College Animal Welfare and Ethical Review Body and the UK Home Office.

### BMDM culture

C57BL/6 mice were obtained from Charles River. Bone marrow cells were isolated from 6–10-wk-old mouse femurs and tibias as previously described (Anand et al., 2011b) and allowed to differentiate into macrophages in DMEM containing 10% heat-inactivated FBS, 1% penicillin/streptomycin, 1% Hepes, and 30% conditioned media from L929 fibroblasts. The cells were allowed to differentiate for 5–6 d at 37°C. After being harvested, cells for some experiments were grown in media containing 5% lipoprotein-deficient serum as indicated. WT, *Nlrp3^−/−^, Asc^−/−^, and Casp1/11^−/−^* iBMDMs were provided by K. Fitzgerald (University of Massachusetts Medical School, Worcester, MA), T.-D. Kanneganti (St. Jude Children's Research Hospital, Memphis, TN), and C. Bryant (University of Cambridge, Cambridge, UK). iBMDMs were cultured in DMEM containing 10% FBS, 1% penicillin/streptomycin, and 10% L929 conditioned medium in 10-cm^2^ tissue culture plates. Cells were grown until confluent and split every 2–3 d.

### Cell stimulations

Primary BMDMs and iBMDMs were seeded in 24-, 12-, or six-well plates at a density of 0.5 × 10^6^, 1 × 10^6^, or 2 × 10^6^ cells per well, respectively. Experiments with U18666a (662015; EMD Millipore) were optimized in different cell types so as to block lysosomal efflux of cholesterol and achieve absolute lysosomal accumulation. Primary BMDMs exposed to U18666a displayed lysosomal cholesterol accumulation at 48 h, while in iBMDMs, sufficient cholesterol accumulation was observed by 24 h (Fig. S1). Where used, cells were exposed to Torin1 (CAY10997; Cayman Chemical) for 3 h, and Baf A1 (Sigma-Aldrich) and CQ (Sigma-Aldrich) at the indicated concentrations for 1 h before ATP stimulation.

### Inflammasome activation assays

For NLRP3 activation, cells were primed with 500 ng/ml of either LPS (tlrl-pbslps; Invivogen) or Pam3 (tlrl-pms; Invivogen) for 4 h followed by addition of either 5 mM ATP (A6419; Sigma-Aldrich) or 20 nM nigericin (4312; Tocris) for 45 min. Cell images were taken using a light microscope following addition of either ATP or nigericin. Alum was used at 1 mg/ml for 16 h in LPS-primed cells grown in complete media. For NLRC4 activation, cells were infected with *S. typhimurium* strain SL1344 (gift from J. Galan [Yale University School of Medicine, New Haven, CT] and A. Shenoy [Imperial College London, London, UK]) at a MOI of 2 for ∼4 h. For AIM2 inflammasome activation, cells grown in 24-well plates were transfected with 1 µg of poly(dA:dT) using Lipofectamine 2000 (Invitrogen) according to the manufacturer’s recommendations. Following each condition, cell supernatants were collected and immediately frozen for cytokine analysis, and the cell lysates were harvested in radioimmunoprecipitation assay (RIPA) lysis buffer for immunoblot analysis.

### Generation of CRISPR-Cas9 targeting constructs

The CRISPR design tool (http://crispr.mit.edu) was used to identify two candidate gRNA sequences (compatible with *Streptococcus pyogenes* Cas9 and its protospacer-adjacent motif [PAM; 5′-NGG]) near the 5′ region of the mouse *Npc1* gene (GenBank Accession no. NM_008720). 5′-phosphorylated oligonucleotides containing the gRNA sequences (underlined) and BbsI adapter are as follows: gRNA1 (5′-CACCGCGACCCGAGCGTCGCGGCAC-3′ and its reverse complement 5′-AAACGTGCCGCGACGCTCGGGTCGC-3′) and gRNA2 (5′-CACCGTCGGAACCGGCGCCTGACCA-3′ and its reverse complement 5′-AAACTGGTCAGGCGCCGGTTCCGAC-3′). The gRNA1 and gRNA2 oligonucleotides were annealed and ligated into pSpCas9(BB)-2A-GFP plasmid (48138; Addgene) at the *BbsI* restriction site as described (Ran et al., 2013). This allows constitutive expression of sgRNAs under the U6 promoter, while *Sp*Cas9 and GFP are expressed by the CBh promoter as two separate proteins due to the intervening self-cleaving T2A peptide. The recombinant plasmids (pSpCas9(BB)-2A-GFP-Npc1_gRNA1 and pSpCas9(BB)-2A-GFP-Npc1_gRNA2) were PCR-screened using the U6 promoter sequence and respective gRNA1 or gRNA2 sequence as a reverse primer. Additionally, they were sequence-verified using a U6 promoter primer (5′-ATTTCTTGGGTAGTTTGCAG-3′).

### Transfection, selection, and validation of *Npc1* mutant lines

iBMDMs were transfected with 500 ng of pSpCas9(BB)-2A-GFP-Npc1_gRNA1 and pSpCas9(BB)-2A-GFP-Npc1_gRNA2 separately using 1.5 µl transfection reagent (Xfect; Takara Bio Inc.) in 600 µl OptiMEM media (Invitrogen) at 37°C. After 6 h, the OptiMEM transfection mixtures were replaced with DMEM growth media supplemented with 10% FBS and 2% penicillin/streptomycin. Cells were incubated at 37°C in 5% CO_2_ for 48 h. The transfected cells were analyzed for expression of GFP (proxy for Cas9 expression) using a fluorescent microscope.

Flow cytometry–based analysis and cell sorting were performed using the FACS Aria II (BD) to select Cas9-expressing cells. 48 h after transfection, the cells were trypsinized, harvested, and resuspended in Sorting Buffer (PBS supplemented with 2 mM EDTA and 0.1% BSA). Cells expressing Cas9 (GFP-positive) were isolated by FACS (single-cell sorting) and expanded in a 96-well plate.

Approximately 2 wk after transfection, genomic DNA of hybridoma cell lines was prepared from typically 10^6^ cells by using the GenElute Mammalian Genomic DNA Miniprep Kit (G1N70; Sigma-Aldrich). PCR amplification was performed with primers flanking the sgRNA targeted region (forward, 5′-CTTCCTGCCTTGCGCACAC-3′, and reverse, 5′-CTGTCCCTGGCTAACTGTGG-3′) with the following cycling conditions: initial denaturation 5 min at 95°C, 35 cycles with denaturation at 95°C (30 s), annealing at 60°C (45 s), elongation at 68°C (45 s), and final elongation at 68°C for 5 min. An aliquot (1:10) of the PCR product was analyzed by agarose gel electrophoresis; the rest of the samples were purified using Monarch PCR & DNA Cleanup Kit (T1030; New England Biolabs, Inc.) and sequenced (Fig. S2). Out of 40 clonal cell lines screened, we found two clonal cell lines that showed deletion in the sgRNA-targeted region in the *Npc1* gene. These two independent cell lines with different deletion mutations (KO#1 and KO#2) were expanded and used for functional analysis and used as *Npc1*-deficient (*Npc1^−/−^*) cells. Since both clonal cell lines gave identical results in inflammasome activation assays (Fig. 2), henceforth, we performed further research using only *Npc1^−/−^* #1 cells.

### Filipin staining

Cells seeded at a density of 4 × 10^5^ cells per well were grown on glass coverslips in a 24-well plate and incubated with U16888A overnight (iBMDMs) or for 48 h (BMDMs) to allow cholesterol accumulation. After desired treatments, cells were fixed in 4% paraformaldehyde for 1 h at room temperature. Cells were then stained with 25 µg/ml filipin (F9765; Sigma-Aldrich) overnight and washed three times with PBS before mounting on glass slides. Images were visualized on an SP5 confocal microscope (Leica Microsystems) using 405-nm excitation lasers and processed using ImageJ (National Institutes of Health).

### Cholesterol supplementation assay

MCD (C4555; Sigma-Aldrich) and cholesterol (C8667; Sigma-Aldrich) complexes were prepared by diluting a 25 mg/ml stock solution of cholesterol 1,000-fold in serum-free DMEM containing 50 µM MCD and incubating at 50°C in a water bath for 2 h. Cells were plated into 12-well plates in the presence or absence of U18666a. On the day of the experiment, media were replaced with serum-free DMEM, and cells were primed with LPS (500 ng/ml) for 3 h before replacing with 37°C prewarmed media containing cholesterol–MCD complexes. Cells were then incubated for the indicated times before addition of 5 mM ATP for 45 min.

### Exposure to statins

BMDMs were seeded into 24- or 12-well plates and incubated overnight in media containing 5% lipid-depleted serum (S181L; Biowest). The following morning, cells were stimulated with LPS (500 ng/ml) for 3 h before addition of fatostatin (40 µM; 4444; Tocris), lovastatin (40 µM; 1530; Tocris), or mevastatin (40 µM; 1526; Tocris) for 1 h. For cholesterol restoration, media were replaced following statin treatment, and cells were recovered for 2 h before addition of cholesterol for 1 h. ATP (5 mM) was then added for 45 min.

### Immunoblotting and antibodies

For immunoblotting of casp-1, NLRP3, ASC, IL-1β, GSDMD, and GAPDH, cells were harvested at indicated time points in cell lysis buffer containing NP-40, DTT, and protease inhibitors (Roche). The samples were boiled at 95°C before resolving them on 12% SDS-PAGE gels. For phosphospecific immunoblotting, cells were exposed to 500 ng/ml LPS for different time points ranging from 30 min to 4 h. Cell lysates were extracted in RIPA lysis buffer containing both protease and phosphatase inhibitors (Roche). Samples were placed on ice for 30 min before being centrifuged at 15,000 *g* for 15 min at 4°C to remove nuclei. The supernatant was then collected, and a 10-µl aliquot was used to measure protein concentration using the Pierce BCA Protein Assay Kit (23227; Thermo Fisher Scientific) according to the manufacturer’s instructions. The protein concentration of each sample was then normalized to 1 µg/ml with RIPA lysis buffer. Equal concentration of protein samples was resolved on 6–15% SDS-PAGE gels and then transferred to nitrocellulose membranes (GE Healthcare). Membranes were blocked for 1 h with 5% milk solution in TBS containing 0.05% Tween-20. Membranes were incubated in primary antibodies at 4°C overnight at the dilutions given below. HRP-conjugated secondary antibodies were used at room temperature for 1 h. Proteins were visualized using the Clarity enhanced chemiluminescence substrate (1705060; Bio-Rad) or the Pierce enhanced chemiluminescence Western nlotting substrate (32209; Thermo Fisher Scientific). Images were taken on a Bio-Rad imager and processed using the manufacturer’s software, Image Lab. The primary antibodies used were as follows: casp-1 (1:2,000; AG-20B-0042-C100; AdipoGen), NLRP3 (1:2,000; AG-20B-0014-C100; AdipoGen), ASC (1:2,000; AG-25B-0006, AL177; AdipoGen), GSDMD (1:1,000; ab209845; Abcam), IL-1β (1:500; 12426; Cell Signaling Technology), AKT (1:1,000; 4691; Cell Signaling Technology), phospho-AKT (1:1,000; 4060; Cell Signaling Technology), p70-S6K1 (1:1,000; 2708; Cell Signaling Technology), phospho–p70-S6K1 (1:1,000; 9234; Cell Signaling Technology), and GAPDH (1:2,500; MA5-15738; Thermo Fisher Scientific). Secondary antibodies were obtained from Thermo Fisher Scientific and used at a dilution of 1:5,000.

### Total and esterified cholesterol measurement

WT and *Npc1^−/−^* iBMDMs or primary mouse BMDMs were seeded into 12-well plates at a density of 10^6^ per well. The following morning, the media were changed to LPDS media, and cells were stimulated with LPS (500 ng/ml) for 4 h. MCD samples were treated with 10 mM MCD for 30 min. Samples were then lysed in 140 µl water for 30 min at 37°C before being used for cholesterol measurement. Total cholesterol was measured using the cholesterol Amplex Red assay kit according to the manufacturer’s recommendations (A12216; Thermo Fisher Scientific). Unesterified cholesterol was measured by a slight modification of the original assay by omitting cholesterol esterase in the working reagent. Esterified cholesterol was calculated by subtracting the unesterfied cholesterol from the total cholesterol levels for each sample tested in triplicate. Values were normalized to protein content, which was determined using a BCA protein assay (23225; Thermo Fisher Scientific).

### PM cholesterol measurement

Cholesterol concentration of the PM was measured by a cholesterol oxidase assay as previously described (Chu et al., 2015). WT and *Npc1^−/−^* iBMDMs were seeded into 24-well plates at a density of 0.5 × 10^6^ per well. Cells were then treated with LPS and MCD as above. The cells were then washed three times with cold PBS followed by three washes with ice-cold assay buffer (310 mM sucrose [S0389; Sigma-Aldrich], 1 mM MgSO_4_ [M2643; Sigma-Aldrich], and 0.5 mM Na_3_PO_4_, pH 7.4 [342483; Sigma-Aldrich]). Cells were incubated with 500 µl assay buffer in the presence or absence of cholesterol oxidase (1 U/ml; C8649; Sigma-Aldrich) for 15 min at 37°C. Next, cells were washed with PBS followed by lysis in 90 µl of water for 30 min at 37°C. Total cholesterol was measured as above with the Amplex Red assay for each sample tested in triplicate. PM cholesterol was calculated by subtracting cholesterol measurements for cells exposed to cholesterol oxidase (intracellular cholesterol) from measurements obtained from cells not exposed to cholesterol oxidase (total cholesterol). Values were normalized to protein content determined as above with a BCA assay.

### RT-qPCR

RNA was isolated using TRIzol (T9424; Sigma-Aldrich) according to the manufacturer’s instructions. The RNA pellet was suspended in 10 µl DNase/RNase-free water (Gibco). RNA concentration was measured in 2 µl RNA by NanoDrop, and 250 ng total RNA was reverse-transcribed into cDNA using the High Capacity cDNA Reverse Transcription kit (4368814; Applied Biosystems). Real-time PCR was performed using gene-specific primers and PowerUp SYBR Green (A25741; Applied Biosystems) and run on an ABI7500 or ABI7900HT (Applied Biosystems) fast real-time PCR instrument. The primers used are listed in Table S1.

### Indirect immunofluorescence

WT and *Npc1^−/−^* cells seeded at a density of 4 × 10^5^ cells per well were grown on glass coverslips in a 24-well plate and exposed to either U16888A or statin as described above. In some samples, MitoTracker Deep Red (Invitrogen) was added in OptiMEM media just before ATP stimulation. After desired treatments, cells were fixed in 4% paraformaldehyde for 30 min at room temperature. Cells were then labeled with the following primary antibodies for 1 h at room temperature: NLRP3 (1:200; AG-20B-0014-C100; AdipoGen), Rabbit ASC (1:100; AG-25B-0006, AL177; AdipoGen), mouse ASC (1:100; 04-147, 2EI-7; EMD Millipore), and calreticulin (1:200; ab92516, EPR3924; Abcam). Secondary antibodies were all obtained from Thermo Fisher Scientific and used according to the manufacturer’s recommendations. Images were visualized and acquired on an SP5 confocal microscope and processed using ImageJ and the LAS AF program.

### ELISA

Cell culture supernatants were measured for IL-1β (88-7013-88; eBioscience), IL-18 (7625; MBL), and TNF-α (88-7324-88; eBioscience) using ELISA kits according to the manufacturer’s instructions.

### LDH assay

LDH was measured in the cell culture supernatant using the Pierce LDH Cytotoxicity Assay Kit (88953; Thermo Fisher Scientific) according to the manufacturer’s instructions.

### Statistical analysis

GraphPad Prism 7.0 software was used for data analysis. Data are represented as mean ± SD or SEM and are representative of experiments done at least three times. Statistical significance was determined by unpaired Student’s *t* test; P < 0.05 was considered statistically significant.

### Online supplemental material

Fig. S1 shows lysosomal cholesterol accumulation upon U18666a treatment and that this does not affect the priming of the NLRP3 inflammasome. Fig. S2 shows the editing of *Npc1* gene by CRISPR-Cas9. Fig. S3 shows reduction in AKT–mTOR signaling upon TLR4 ligation when NPC1 function is abrogated. Fig. S4 shows regulation of SREBP2 and PM cholesterol levels in *Npc1^−/−^* cells. Fig. S5 shows the localization of NLRP3 and ASC speck in WT macrophages. Table S1 shows primers used for RT-qPCR.

## Supplementary Material

Supplemental Materials (PDF)

Table S1 (Excel)
