# Supplementary material for: Trafficking of cholesterol to the ER is required for NLRP3 inflammasome activation
Source: J Cell Biol. 2018 Oct 1;217(10):3560–76. doi: 10.1083/jcb.201709057 (PMC6168277; doi:10.1083/jcb.201709057)
Supplement: Supplemental Materials (PDF) [file JCB_201709057_sm.pdf]

Supplemental material

de la Roche et al., https://doi.org/10.1083/jcb.201709057

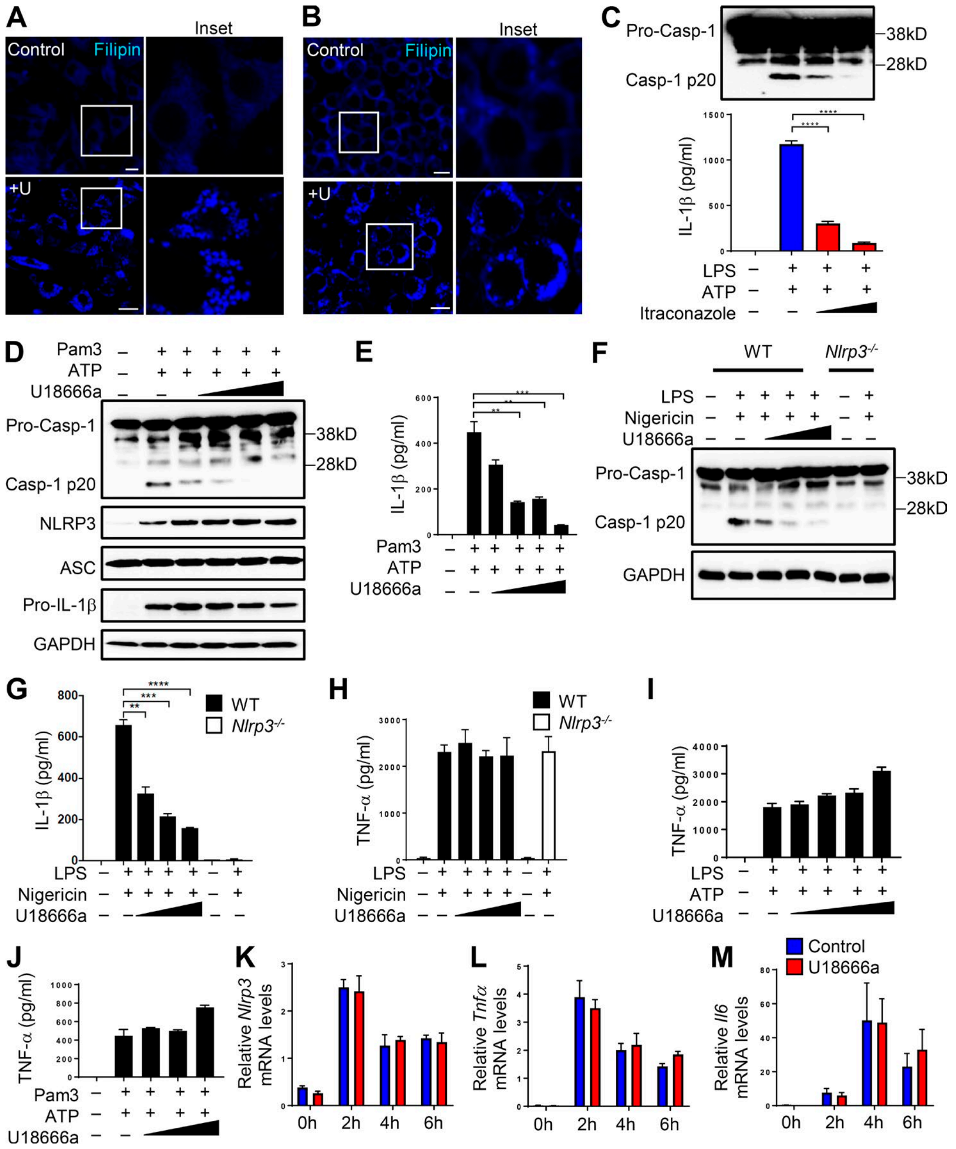

Figure S1. **U18666a treatment results in lysosomal cholesterol accumulation and does not affect the priming step of the NLRP3 inflammasome. (A)** Primary BMDMs grown on coverslips were either left untreated (control) or treated with 5 µg/ml U18666a (+U) for 48 h before staining them with filipin (25 µg/ml). **(B)** iBMDMs grown on coverslips were either left untreated (control) or treated with 5 µg/ml U18666a (+U) overnight before staining them with filipin (25 µg/ml). Note the punctate structures in U18666a-treated cells in A and B showing lysosomal cholesterol accumulation. Bars, 5 µm. **(C)** Primary BMDMs were either left untreated or exposed to increasing concentrations of itraconazole (1 µg/ml and 2 µg/ml) before LPS (500 ng/ml; 4 h) and ATP (5 mM; 45 min) stimulation. Cell lysates were immunoblotted for casp-1, and cell supernatants were analyzed for IL-1β by ELISA. **(D and E)** Primary BMDMs were either left untreated or exposed to increasing concentrations of U18666a (1, 2, 5, and 10 µg/ml) followed by Pam3 (500 ng/ml; 4 h) and ATP (5 mM; 45 min) stimulation. Cell lysates were immunoblotted for the antibodies indicated (D), and cell supernatants were analyzed for IL-1β by ELISA (E). **(F–H)** WT and *Nlrp3*$^{-/-}$ iBMDMs were either left untreated or exposed to increasing concentrations of U18666a (1, 2, and 5 µg/ml) before stimulating them with LPS (500 ng/ml; 4 h) and nigericin (20 µM; 45 min). Cell lysates were immunoblotted for casp-1, and GAPDH as loading control (F). Cell supernatants were analyzed for IL-1β (G) and TNF-α (H) by ELISA. **(I)** BMDMs were either left untreated or exposed to increasing concentrations of U18666a (1, 2, 5, and 10 µg/ml) followed by LPS (500 ng/ml; 4 h) and ATP (5 mM; 45 min) stimulation. TNF-α release in cells supernatants was measured by ELISA. **(J)** BMDMs were treated as in D, and TNF-α release was measured by ELISA. **(K–M)** BMDMs were either left untreated (control) or exposed to 5 µg/ml U18666a before stimulating cells with LPS (500 ng/ml). RNA samples were collected at different times after LPS stimulation, and expression of genes coding for *Nlrp3*, *Tnf*, and *Il-6* was evaluated by real-time qPCR. Bar graphs show levels of target genes relative to controls and are normalized to *Gapdh*. Data shown are mean ± SD, and experiments shown are representative of at least three independent experiments.**, P < 0.01; ***, P < 0.001; ****, P < 0.0001, by Student's *t* test.

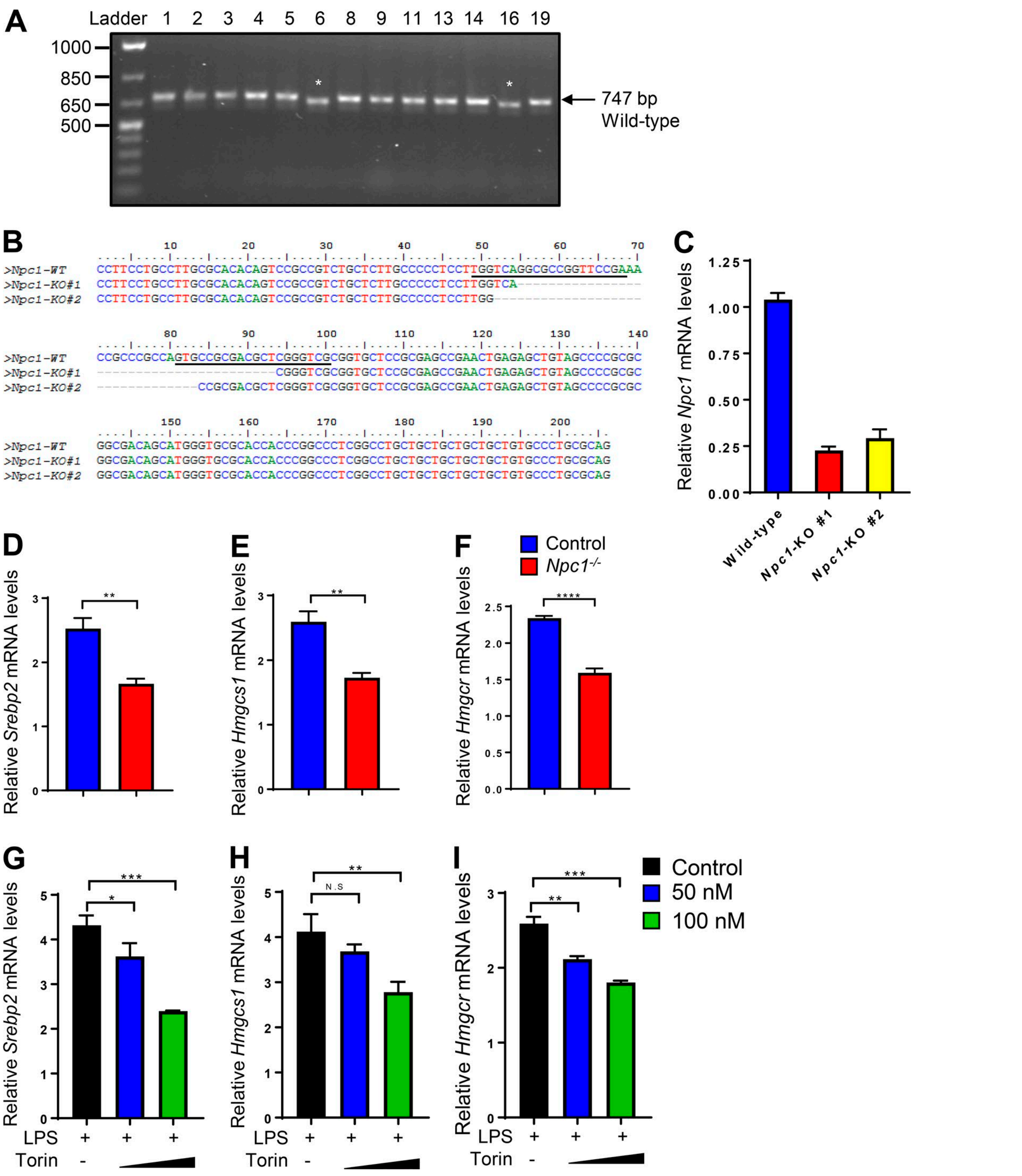

Figure S2. **Editing of *Npc1* gene by CRISPR-Cas9 and reduced SREBP2 activation in *Npc1*$^{-/-}$ cells. (A)** Agarose gel showing DNA fragments generated by PCR amplification of the region targeted by sgRNAs using flanking primer pair. Each lane shows an individual clonal cell line. Note that two of the clonal cell lines indicated by asterisks show bands with a smaller amplicon size, suggesting deletion and correspond with KO#1 (lane 6) and KO#2 (lane 16) cell line, respectively **(B)** Schematic diagram showing Sanger sequencing of the DNA fragment obtained in A above from WT (*Npc1*-WT) and two *Npc1*-deficient (*Npc1*-KO#1 and *Npc1*-KO#2) cell lines. The sequence of the targeting sgRNAs are underlined in *Npc1*-WT. The *Npc1*-KO#1 was generated using gRNA 1 (nt 81–100), while *Npc1*-KO#2 was obtained from gRNA2 (nt 49–68) containing plasmids. Deleted sequences in *Npc1*-deficient (*Npc1*-KO#1 and *Npc1*-KO#2) cell lines are shown by dashed lines. **(C)** *Npc1* transcript expression in LPS-primed WT (*Npc1*-WT), and two *Npc1*-deficient (*Npc1*-KO#1 and *Npc1*-KO#2) cell lines. Bar graph shows levels of *Npc1* mRNA in *Npc1*-KO cell lines relative to the WT and are normalized to *Gapdh*. **(D–F)** WT and *Npc1*$^{-/-}$ were stimulated with LPS (500 ng/ml). RNA samples were collected at 4 h after LPS stimulation, and expression of genes coding for *Srebp2*, *Hmgcs1*, and *Hmgcr* was quantified by real-time PCR. Bar graphs show levels of target genes relative to control untreated cells (control) and are normalized to *Gapdh*. **(G–I)** BMDMs were exposed to mTOR inhibitor Torin1 (50 nM and 100 nM; 2 h) before stimulating them with LPS (500 ng/ml). RNA samples were collected at 4 h after LPS stimulation, and expression of genes coding for *Srebp2*, *Hmgcs1*, and *Hmgcr* was quantified by real-time PCR. Bar graphs show levels of target genes relative to *Gapdh*. Data shown are mean ± SD, and experiments shown are representative of at least three independent experiments. *, P < 0.05; **, P < 0.01; ***, P < 0.001; ****, P < 0.0001, by Student's *t* test.

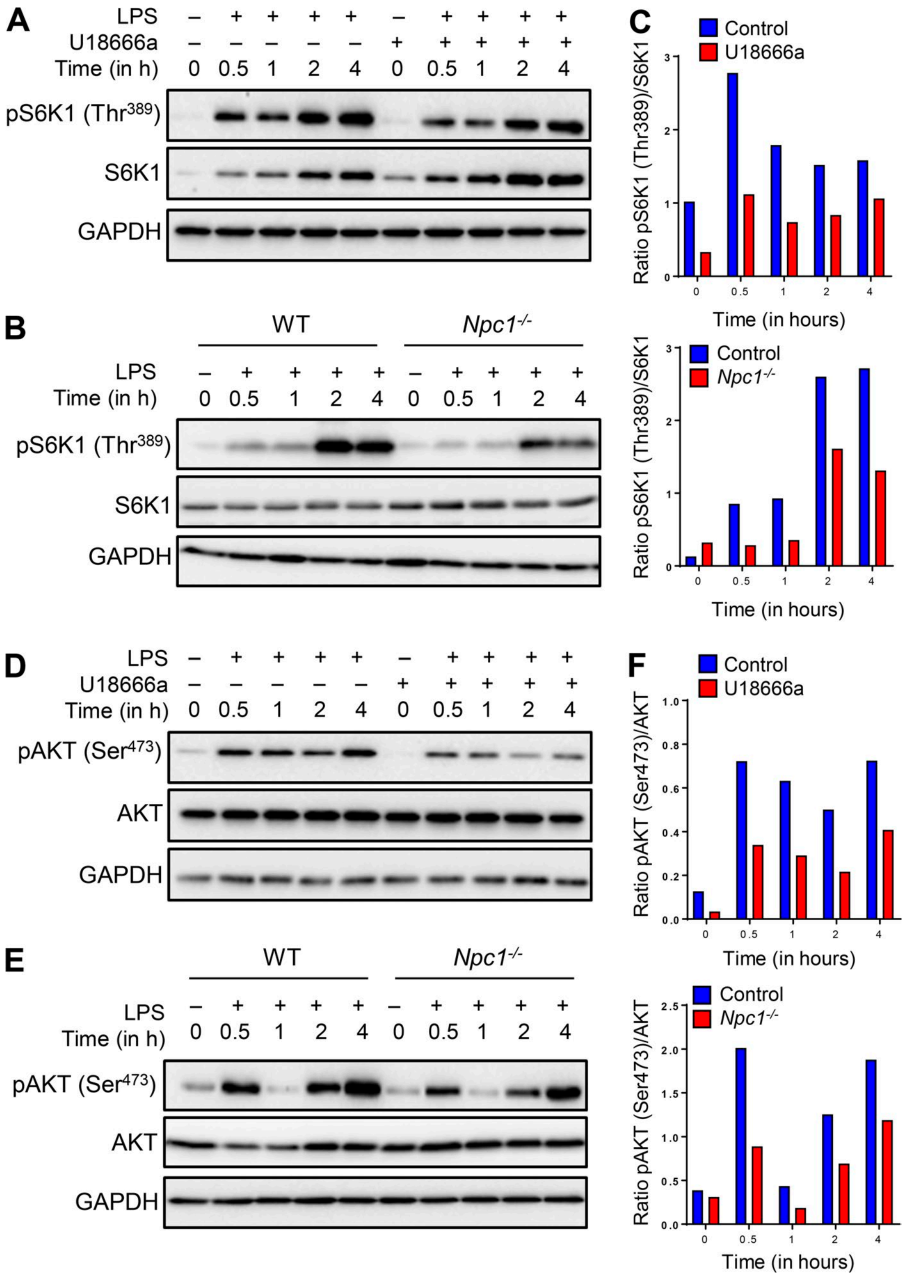

Figure S3. **Blockade of NPC1 function disengages the AKT–mTOR pathway. (A and D)** BMDMs were either left untreated or exposed to 5 µg/ml U18666a before stimulating with LPS (500 ng/ml) for the indicated times. Samples were immunoblotted with the indicated antibodies. GAPDH was used as a loading control. **(B and E)** WT and *Npc1*$^{-/-}$ cells were stimulated with LPS (500 ng/ml) for indicated times. Samples were immunoblotted with the indicated antibodies. GAPDH was used as a loading control. **(C and F)** Bar graphs showing semiquantification of phosphorylated S6K1 and phosphorylated AKT levels relative to total S6K1 and total AKT levels, respectively.

https://doi.org/10.1083/jcb.201709057

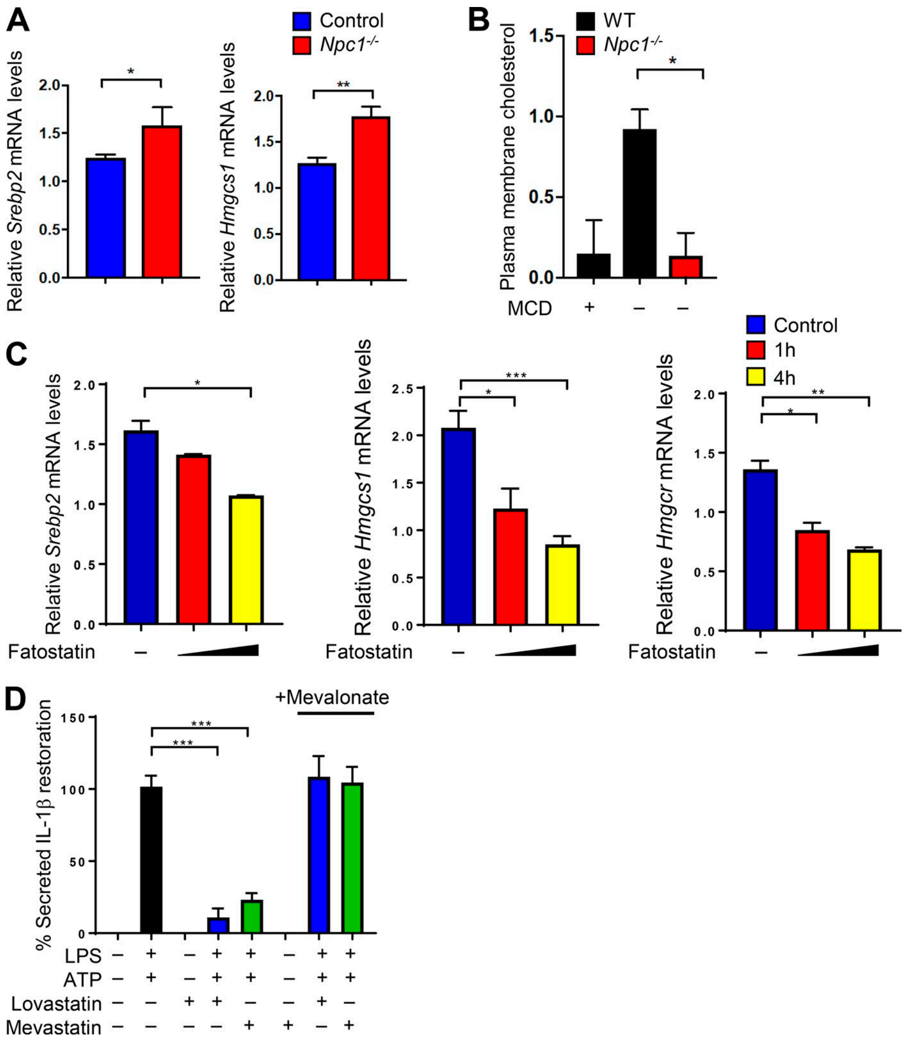

Figure S4. **Regulation of SREBP2 and PM cholesterol levels in *Npc1*$^{-/-}$ cells. (A)** RNA from control unstimulated WT and *Npc1*$^{-/-}$ was collected, and expression of genes coding for *Srebp2 and Hmgcs1* was quantified by real-time PCR. Bar graphs show levels of target genes normalized to *Gapdh*. **(B)** PM cholesterol levels in WT cells either exposed or not to MCD (10 mM, 30 min) and *Npc1*$^{-/-}$ cells. **(C)** WT iBMDMs grown overnight in lipid-depleted media were exposed to SREBP inhibitor fatostatin (40 µM). RNA samples were collected at indicated times, and expression of genes coding for *Srebp2*, *Hmgcs1*, and *Hmgcr* was quantified by real-time PCR. Bar graphs show levels of target genes relative to *Gapdh*. **(D)** Secreted IL-1β levels upon addition of mevalonate for 4 h before ATP stimulation from BMDMs treated as in Fig. 5 G. Data shown are mean ± SD, and experiments shown are representative of at least three independent experiments. *, $P < 0.05$; **, $P < 0.01$; ***, $P < 0.001$, by Student's *t* test.

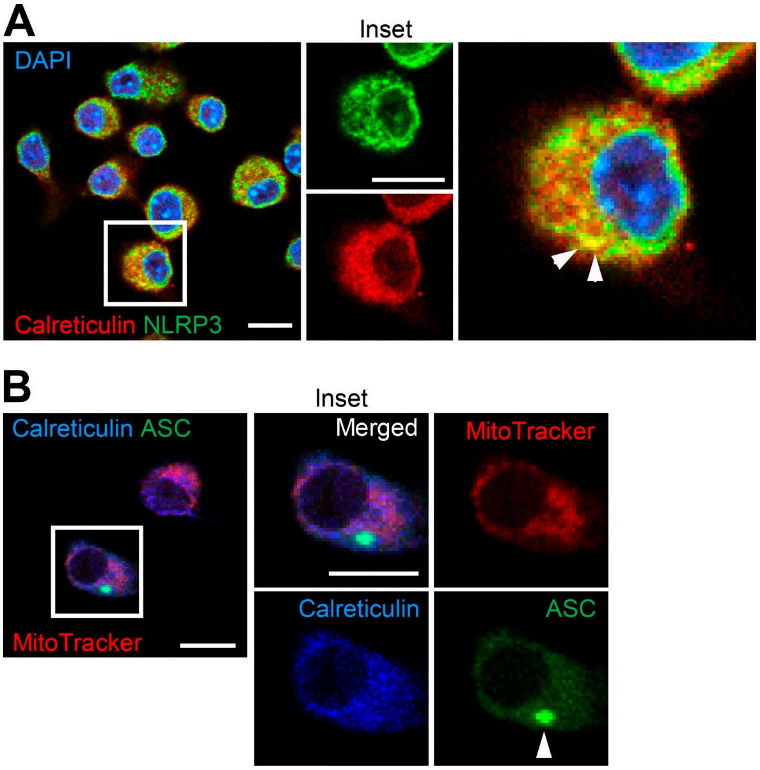

Figure S5. **Localization of NLRP3 and ASC speck. (A)** WT BMDMs were exposed to LPS and subsequently fixed and labeled for NLRP3 and ER marker calreticulin with secondary antibodies conjugated to FITC and Alexa Fluor 546, respectively. Inset and right panel show magnifications of indicated regions on the left panel showing merged image. Arrowheads depict association of NLRP3 and calreticulin. **(B)** WT BMDMs were stimulated with LPS and exposed to MitoTracker Deep Red (Alexa Fluor 647) during ATP stimulation in OptiMEM. Subsequently, cells were fixed and labeled for endogenous ASC and ER marker calreticulin with secondary antibodies conjugated to FITC and Alexa Fluor 405, respectively. Inset shows zoom of indicated region on the left panel. Arrowhead on the bottom right panel shows an ASC speck. Bars, 10 µm. Data shown are representative of at least three independent experiments.

Table S1 is a separate Excel document showing primers used for real-time qPCR.
